# Angiotensin Converting Enzyme (ACE) Inhibitor Extends *Caenorhabditis elegans* Life Span

**DOI:** 10.1371/journal.pgen.1005866

**Published:** 2016-02-26

**Authors:** Sandeep Kumar, Nicholas Dietrich, Kerry Kornfeld

**Affiliations:** Department of Developmental Biology, Washington University School of Medicine, St. Louis, Missouri, United States of America; Stanford University Medical Center, UNITED STATES

## Abstract

Animal aging is characterized by progressive, degenerative changes in many organ systems. Because age-related degeneration is a major contributor to disability and death in humans, treatments that delay age-related degeneration are desirable. However, no drugs that delay normal human aging are currently available. To identify drugs that delay age-related degeneration, we used the powerful *Caenorhabdtitis elegans* model system to screen for FDA-approved drugs that can extend the adult lifespan of worms. Here we show that captopril extended mean lifespan. Captopril is an angiotensin-converting enzyme (ACE) inhibitor used to treat high blood pressure in humans. To explore the mechanism of captopril, we analyzed the *acn-1* gene that encodes the *C*. *elegans* homolog of ACE. Reducing the activity of *acn-1* extended the mean life span. Furthermore, reducing the activity of *acn-1* delayed age-related degenerative changes and increased stress resistance, indicating that *acn-1* influences aging. Captopril could not further extend the lifespan of animals with reduced *acn-1*, suggesting they function in the same pathway; we propose that captopril inhibits *acn-1* to extend lifespan. To define the relationship with previously characterized longevity pathways, we analyzed mutant animals. The lifespan extension caused by reducing the activity of *acn-1* was additive with caloric restriction and mitochondrial insufficiency, and did not require *sir-2*.*1*, *hsf-1* or *rict-1*, suggesting that *acn-1* functions by a distinct mechanism. The interactions with the insulin/IGF-1 pathway were complex, since the lifespan extensions caused by captopril and reducing *acn-1* activity were additive with *daf-2* and *age-1* but required *daf-16*. Captopril treatment and reducing *acn-1* activity caused similar effects in a wide range of genetic backgrounds, consistent with the model that they act by the same mechanism. These results identify a new drug and a new gene that can extend the lifespan of worms and suggest new therapeutic strategies for addressing age-related degenerative changes.

## Author Summary

Age-related degeneration is a fundamental feature of animal biology and an important contributor to human disability and death. However, no medicines have been shown to delay human aging. To identify drugs that delay age-related degeneration, we screened FDA-approved compounds and discovered that the hypertension drug captopril significantly extended *C*. *elegans* lifespan. In humans, captopril inhibits angiotensin converting enzyme (ACE) to regulate blood pressure. The *C*. *elegans* homolog of ACE is encoded by the *acn-1* gene. We discovered that reducing the activity of *acn-1* also caused a robust extension of lifespan and delayed age-related changes in *C*. *elegans*. Captopril and *acn-1* have a similar mechanism of action; both treatments displayed similar interactions with previously characterized pathways, and combining treatment with captopril and reducing the activity of *acn-1* did not have an additive effect on life span extension. These results identify a new drug and a new gene that influence aging in *C*. *elegans*. They may be relevant to other animals such as humans because the pathway that includes ACE has been conserved during evolution. These findings establish a foundation for possible therapeutic interventions that can delay age-related degeneration.

## Introduction

Animal aging is characterized by progressive, degenerative changes of tissue structure and function. In humans, these changes have profound negative effects on health by causing morbidity and mortality. An important goal of aging research is to identify interventions that can delay age-related degeneration and promote an extended period of vitality or healthspan. However, no interventions have been demonstrated to delay human aging. By contrast, a growing number of interventions have been demonstrated to delay age-related degeneration and extend lifespan in model animals such as worms, flies and mice [1]. These interventions include dietary changes such as caloric restriction, genetic changes such as reducing the activity of the insulin/insulin-like growth factor-1 (IGF-1) signaling pathway, and drugs such as rapamycin. These studies indicate that pathways that influence aging have been conserved during animal evolution [1]. Thus, model organisms are promising systems to identify and characterize interventions that promote healthy aging and may be beneficial in humans.

The terrestrial nematode *Caenorhabditis elegans* has emerged as an outstanding model organism for studies of aging. The biology of these animals is well suited for studies of aging because they have a rapid life cycle and a relatively short adult lifespan of about 15 days [2,3]. A wide variety of age-related degenerative changes have been documented, providing assays of aging and suggesting *C*. *elegans* undergoes mechanisms of aging similar to larger animals where progressive degenerative changes are well characterized [4]. Powerful experimental techniques are well established, including forward and reverse genetic approaches and molecular approaches facilitated by a fully sequenced genome [5,6]. *C*. *elegans* are well suited for pharmacological studies because they ingest compounds that are added to the culture medium. Molecular genetic studies have identified and characterized several pathways that substantially influence the rate of age-related degeneration. The insulin/IGF-1 pathway was first implicated in aging biology in *C*. *elegans* and has now been shown to play a conserved role in other animals, including flies and mammals [1]. Mutations that reduce the activity of the *daf-2* insulin receptor or the *age-1* phosphatidylinositol-3-OH (PI3) kinase substantially extend the adult lifespan, indicating that insulin/IGF-1 pathway activity promotes a rapid lifespan [7,8]; these mutant animals also display enhanced resistance to a variety of stresses such as UV light, oxidation, transition metals, and hypoxia [9–12]. A critical effector of the *daf-2/age-1* pathway is the forkhead transcription factor DAF-16, which is activated and localized to the nuclei by low levels of *daf-2* signaling [13,14]. The activity of *daf-16* promotes an extended lifespan, and *daf-16* is necessary for the extension of lifespan caused by mutations of *daf-2* and *age-1* [8,15]. Caloric restriction extends the lifespan of a wide range of organisms, including *C*. *elegans*, indicating that *ad libitum* feeding promotes a rapid lifespan. Mutations of genes that are necessary for pharyngeal pumping and food ingestion, such as *eat-2*, cause a substantial lifespan extension [16]. Mutations in multiple genes that are necessary for mitochondrial function, such as *isp-1*, cause a lifespan extension, indicating that wild-type levels of mitochondrial activity promote a rapid lifespan [17,18]. In addition to genetic approaches, *C*. *elegans* is emerging as a valuable system for pharmacological approaches that can be used to identify and characterize drugs that influence aging. Compounds that influence *C*. *elegans* aging have been identified by screening approaches and by testing candidate drugs based on a known mechanism of action [19–25].

To identify drugs that influence aging, we screened FDA-approved drugs for the ability to extend the lifespan of *C*. *elegans* hermaphrodites. Here we report that captopril, an angiotensin converting enzyme (ACE) inhibitor used to treat high blood pressure, extended mean lifespan. ACE is a protease that functions in a signaling cascade that is initiated by low blood pressure; in humans, ACE converts angiotensin I to angiotensin II, and angiotensin II binds the AT1 receptor, resulting in increased contractility of endothelial cells and thereby increasing blood pressure [26,27]. ACE inhibitors such as captopril are used by a large number of people to control hypertension [27]. The ACE gene has been conserved from bacteria to mammals, indicating it had a primordial function before the evolution of a closed circulatory system that creates blood pressure. The *C*. *elegans* homolog of ACE is encoded by the *acn-1* gene; *acn-1* is necessary for larval molting but has not been previously implicated in adult longevity [28]. We hypothesized that captopril inhibits *acn-1* to extend lifespan, and here we present experimental evidence that supports this model. First, inhibition of the *acn-1* gene by RNA interference extended lifespan and delayed age-related degenerative changes, indicating that *acn-1* activity influences aging and longevity. Second, captopril treatment and reducing the activity of *acn-1* caused very similar effects in a wide range of genetic backgrounds, indicating that these interventions have a common mechanism. Third, the lifespan extensions caused by captopril treatment and reducing the activity of *acn-1* were not additive, indicating that these interventions may affect the same pathway. These results identify captopril as a new, FDA-approved drug that can extend the lifespan of *C*. *elegans* and *acn-1* as a new gene that influences *C*. *elegans* aging. The findings establish *acn-1* as the target of captopril in worms, connecting a pharmacological intervention that extends lifespan to its direct molecular target. In mammals, ACE regulates blood pressure, indicating there is a link between a system that controls aging in worms and physiology in mammals.

## Results

### Captopril extended *C*. *elegans* lifespan

**Fig 1:**
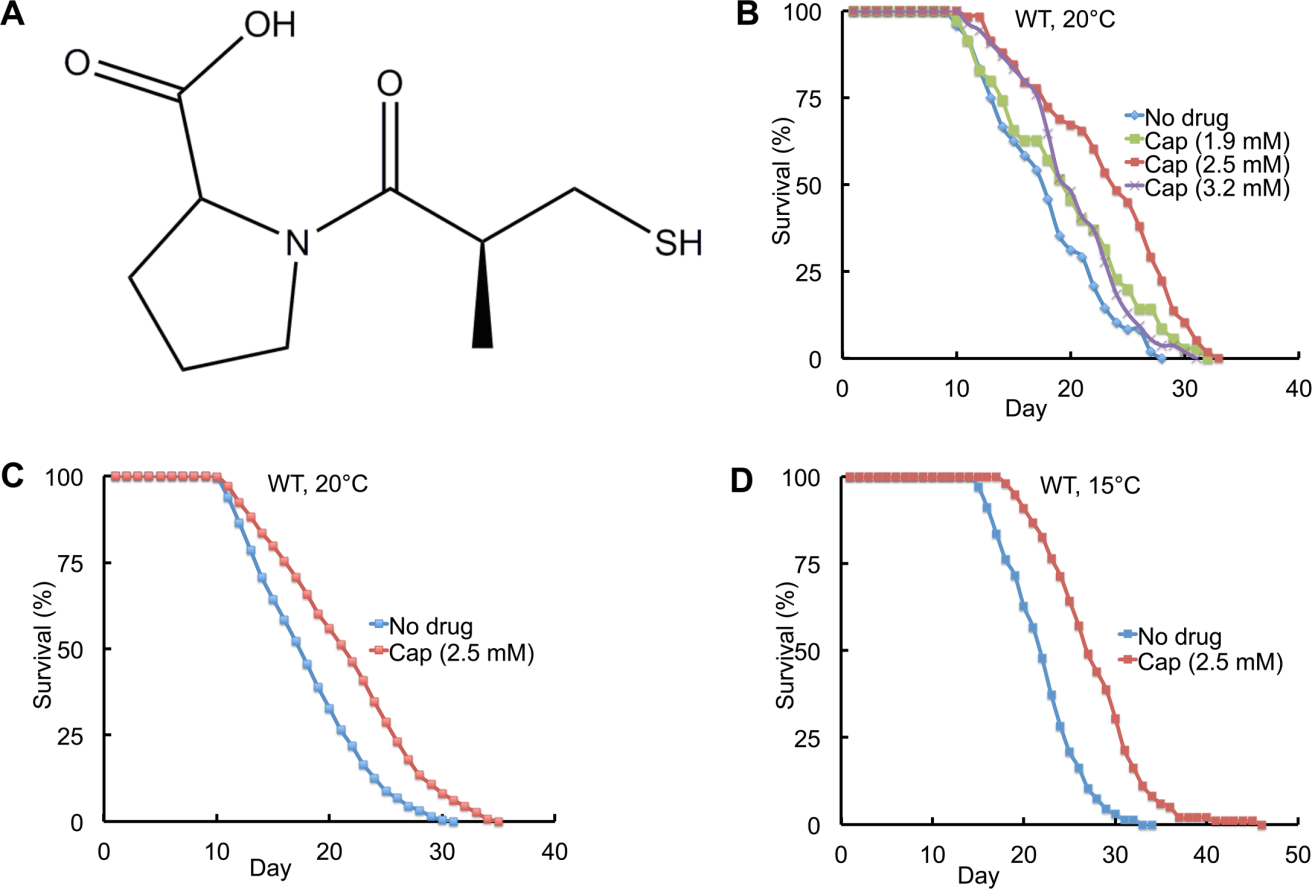
Captopril extended the adult lifespan. (A) Structure of captopril. (B) Survival curves of wild-type (WT) hermaphrodites cultured at 20°C with no drug or captopril (Cap) at concentrations of 1.9 mM, 2.54 mM and 3.18 mM in the NGM medium. Hermaphrodites were exposed to captopril starting at the L4 stage (day 0) and monitored regularly until death. (C) Wild-type hermaphrodites were treated with no drug or 2.54 mM captopril at 20°C. These data represent the analysis of 500 animals in 10 trials, whereas the data in panel B represent 58 animals in 2 trials. (D) WT hermaphrodites were cultured at 15°C. See Table 1 for summary statistics, number of animals and number of independent experiments.

To identify drugs that influence aging, we selected 15 compounds that are Food and Drug Administration (FDA)-approved for human use, have known effects on human physiology, and represent different functional or structural classes (see Materials and Methods). Compounds were added to NGM agar at three different concentrations, and the lifespan of *C*. *elegans* hermaphrodites cultured at 20°C with *E*. *coli* OP50 as a food source was determined. We previously described a similar screening approach that was used to identify the lifespan extending compounds ethosuximide and valproic acid [19,20]. Captopril, an ACE inhibitor, caused a significant extension of lifespan (Fig 1A and 1C). To identify the optimal concentration for lifespan extension, we performed a dose–response analysis. A concentration of 2.5mM captopril in the medium caused the greatest lifespan extension, whereas concentrations of 1.9mM and 3.2mM caused smaller extensions (Fig 1B; Table 1, line 1–4). At the optimal concentration of 2.5mM, captopril treatment caused a significant 23% extension of mean adult lifespan and a significant 18% extension of maximum adult lifespan (Fig 1C; Table 1, line 5–6). We define maximum adult lifespan as the average lifespan of the 10% of the population that are longest lived. To determine the developmental stage when captopril functions to extend lifespan, we administered the drug beginning at the L4 larval stage. The drug was effective with this time of administration suggesting captopril functions in adults to delay age-related degeneration. To determine the temperature dependence of captopril, we analyzed animals cultured at 15°C, 20°C and 25°C. Captopril significantly extended the mean and maximum adult lifespan at all three temperatures, indicating that the effect is not temperature dependent (Fig 1D; Table 1, line 7–10; S1A Fig).

**Table 1:** Captopril extended adult lifespan.

Line	Genotype^1^	Drug^2^	Mean^3^ lifespan ± SD (days)	% Change	Maximum^3^ lifespan ± SD (days)	% Change	N (n)^4^
1	WT	None	15.9±5.0		24.9±1.0		48 (2)
2	WT	Captopril (1.9 mM)	17.7±6.1^ns^	+11	27.9±1.7*	+11	35 (2)
3	WT	Captopril (2.54 mM)	21.4±4.1***	+34	29.4±0.8***	+16	58 (2)
4	WT	Captopril (3.18 mM)	18.4±5.2*	+18	26.2±1.5^ns^	+5	54 (2)
5	WT	None	16.2±4.9		25.2±1.6		531 (10)
6	WT	Captopril (2.54 mM)	19.9±6.1***	+23	29.8±1.5***	+18	500 (10)
7	WT 15°C	None	20.3±4.1		27.4±1.7		67 (2)
8	WT 15°C	Captopril (2.54 mM)	25.9±5.2***	+28	36.2±3.7***	+32	98 (2)
9	WT 25°C	None	7.9±2.7		13.3±1.4		112 (3)
10	WT 25°C	Captopril (2.54 mM)	10.0±3.8**	+22	15.5±1.4**	+16	77 (3)
11	WT (dead OP50)	None	17.3±4.2		24.6±1.0		117 (3)
12	WT (dead OP50)	Captopril (2.54 mM)	20.4±5.0***	+18	28.3±0.9***	+15	111 (3)
13	*eat-2(ad1116)*	None	17.9±5.1		26.2±6.1		117 (3)
14	*eat-2(ad1116)*	Captopril (2.54 mM)	20.4±1.1***	+14	30.7±1.4***	+17	122 (3)
15	*isp-1(qm150)*	None	20.0±6.4		30.9±2.7		142 (3)
16	*isp-1(qm150)*	Captopril (2.54 mM)	24.6±7.7***	+23	37.1±2.2***	+20	132 (3)
17	*sir-2*.*1(ok434)*	None	17.0±3.7		23.42±1.2		188 (3)
18	*sir-2*.*1(ok434)*	Captopril (2.54 mM)	19.8±4.9***	+17	29.7±3.6**	+27	110 (3)
19	*daf-16(mu86)*	None	12.6±3.5		18.4±1.7		200 (3)
20	*daf-16(mu86)*	Captopril (2.54 mM)	11.3±2.7***	-10	16.4±1.7***	-11	202 (3)
21	*daf-2(e1370)*	None	35.4±8.9		49.8±3.0		157 (4)
22	*daf-2(e1370)*	Captopril (2.54 mM)	40.3±9.3***	+11	54.9±3.0**	+9	184 (4)

^1^Genotype: Wild-type hermaphrodites or the indicated mutants were analyzed at 20°C, except those labeled 15°C or 25°C. Animals were cultured with live *E*. *coli* OP50, except those labeled dead OP50, which were cultured with UV light killed bacteria.

^2^Drug: Animals were cultured on standard NGM (None) or on NGM containing the indicated concentration of captopril.

^3^Mean, Maximum, % Change: Maximum adult lifespan is the mean lifespan of the 10% of the population that had the longest lifespans. Comparisons are to the paired no drug treatment control

*, *P* < 0.05

**, *P* < 0.005

***, *P* < 0.0001

n.s., not significant, P>0.05.

^4^N(n): Total number of hermaphrodites analyzed, and the number of independent experiments.

These experiments were conducted with live *E*. *coli* as a food source, raising the possibility that captopril may directly affect bacteria and indirectly affect worms. There are precedents for such a mechanism, since antibiotics can extend *C*. *elegans* lifespan by reducing the pathogenicity of bacteria [29–31], and the anti diabetic drug metformin was reported to extend *C*. *elegans* lifespan by altering bacterial folate and methionine metabolism [32]. To determine if the effects of captopril are mediated by an effect on live bacteria, we conducted the life span experiment with bacteria killed by exposure to ultraviolet light. Captopril extended the lifespan of C. *elegans* in these conditions, demonstrating that the mechanism of captopril-mediated lifespan extension is not dependent on live E. *coli* (S1C Fig; Table 1, line 11–12).

A large number of age-related degenerative changes have been characterized in *C*. *elegans*, including declines of physiological processes, such as body movement, pharyngeal pumping, and egg-laying, and changes in morphology, such as loss of tissue integrity [4,33,34]. Treatment with captopril caused a small delay in the age-related decline in pharyngeal pumping rate, although the change was not statistically significant with the sample size analyzed (S2 Fig). Several genetic manipulations that extend adult lifespan also affect reproduction. For example, caloric restriction and defects in insulin/IGF-1 signaling reduce total progeny production and increase reproductive span in self-fertile hermaphrodites [35]. To determine how captopril affects reproduction, we monitored progeny production of self-fertile hermaphrodites daily. Captopril did not significantly affect total brood size or reproductive span of self-fertile hermaphrodites (S3A and S3B Fig).

### Reducing the activity of *acn-1* extended lifespan

**Fig 2:**
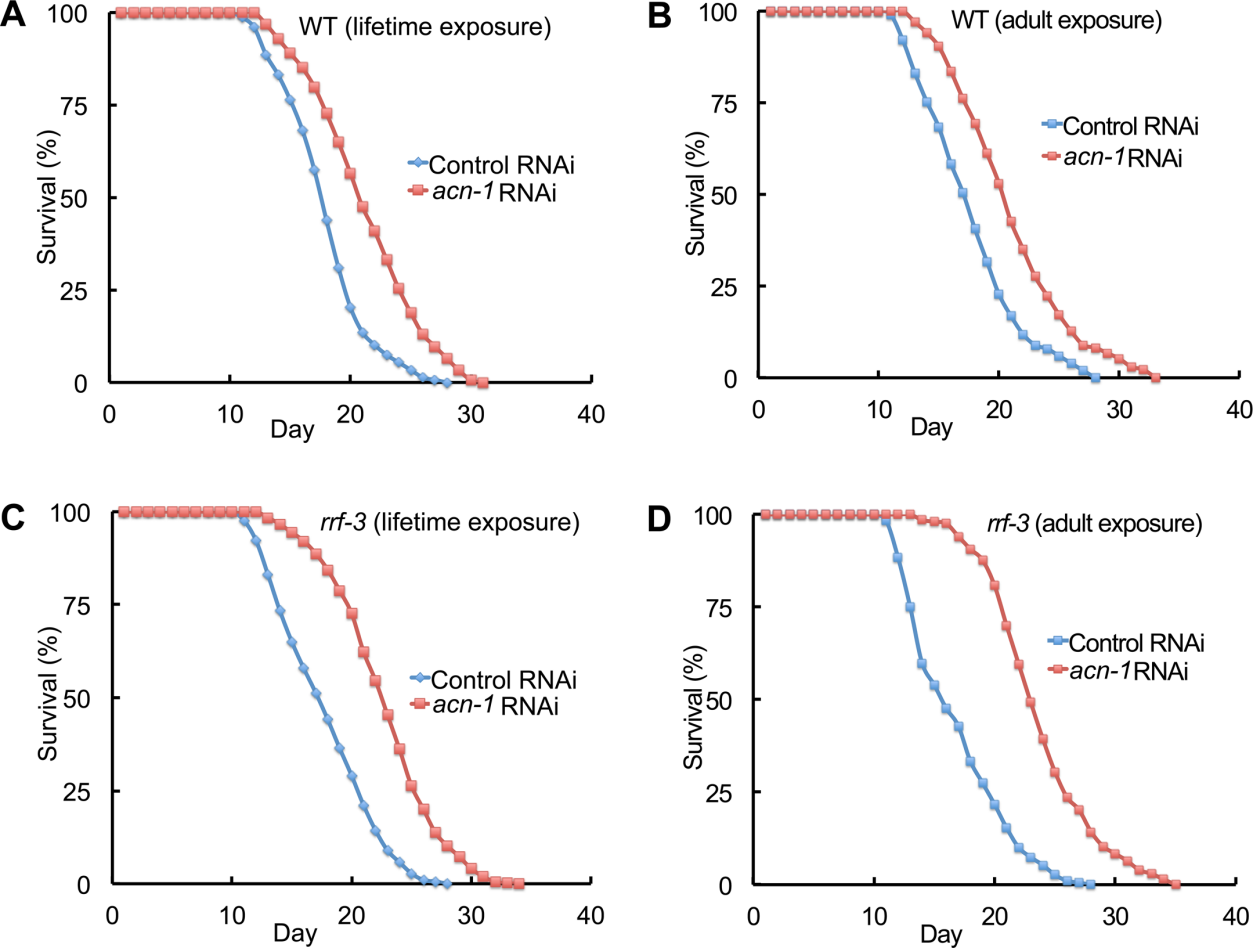
*acn-1* RNAi extended the adult lifespan. Survival curves of wild-type (WT) hermaphrodites (A, B) and *rrf-3* mutant hermaphrodites (C, D) cultured at 20°C with bacteria containing the control RNAi plasmid (L4440, blue) or the *acn-1* RNAi plasmid (red). Hermaphrodites were exposed to RNAi bacteria starting at the embryonic stage (A, C, lifetime exposure) or the L4 stage (B, D, adult exposure). See Table 2 for summary statistics, number of animals and number of independent experiments.

Captopril treatment in humans reduces blood pressure by inhibiting the activity of angiotensin converting enzyme (ACE) [36]. Therefore, we hypothesized that captopril treatment in *C*. *elegans* extends longevity by inhibiting the worm homolog of ACE. To investigate this hypothesis, we analyzed the *acn-1* gene because it encodes a predicted protein that is most similar to human ACE [28]. To reduce the activity of *acn-*1, we used RNA interference (RNAi) [37]; worms were fed bacteria expressing dsRNA from the *acn-1* gene, which is predicted to reduce the levels of the *acn-1* transcript. Wild-type animals cultured with *acn-1* RNAi beginning at the embryonic stage displayed a significant extension of mean and maximum lifespan of 21% and 18%, respectively (Fig 2A, Table 2, line 1–2). These results indicate that *acn-1* activity is necessary to promote a rapid lifespan. To investigate the time of action of *acn-1*, we initiated the exposure to *acn-1* RNAi at the L4 larval stage. Exposure only during adulthood caused a similar extension of mean and maximum lifespan of 22% and 20%, respectively, indicating that *acn-1* functions in adults to promote a rapid lifespan (Fig 2B, Table 2, line 3–4).

**Table 2:** *acn-1* RNAi extended adult lifespan.

Line	Genotype^1^	RNAi^2^	Mean^3^ lifespan ± SD (days)	% Change	Maximum^3^ lifespan ± SD (days)	% Change	N (n)^4^
1	WT	Control	16.0±3.5		22.7±1.6		148 (3)
2	WT	*acn-1*	19.3±4.4***	+21	26.8±1.0***	+18	154 (3)
3	WT	Control (adult)	15.8±4.0		23.5±1.7		101 (2)
4	WT	*acn-1* (adult)	19.2±4.7***	+22	28.2±2.0***	+20	134 (2)
5	*rrf-3(pk1426)*	Control	15.7± 4.0		22.7±1.2		503 (11)
6	*rrf-3(pk1426)*	*acn-1*	20.9±4.2***	+33	28.1±1.2***	+24	620 (11)
7	*rrf-3(pk1426)*	Control (adult)	14.9± 4.0		22.5± 1.4		189 (3)
8	*rrf-3(pk1426)*	*acn-1* (adult)	21.8±4.3***	+46	29.8± 1.7***	+33	203 (3)
9	*rrf-3(pk1426)*	Control (25°C)	8.3± 1.2		10.4± 0.5		50 (1)
10	*rrf-3(pk1426)*	*acn-1* (25°C)	9.9±1.9***	+20	13.6± 0.5***	+31	50 (1)
11	*eat-2(ad1116)*	Control	18.2±4.8		26.9±2.2		171 (3)
12	*eat-2(ad1116)*	*acn-1*	20.3±5.1***	+12	29.4±1.9***	+10	231 (4)
13	*isp-1(qm150)*	Control	23.3±6.2		33.2±1		199 (3)
14	*isp-I(qm150)*	*acn-1*	26.4±7.4***	+14	37.3±2***	+13	151 (3)
15	*sir-2*.*1(ok434)*	Control	14.8±2.8		19.8±1.0		203 (3)
16	*sir-2*.*1(ok434)*	*acn-1*	17.5±3.1***	+16	23.0±1.3***	+18	214 (3)
17	*rict-1(mg360)*	Control	12.1±2.6		16.7±0.8		113 (2)
18	*rict-1(mg360)*	*acn-1*	14.9±3.0***	+23	20.9±11.3***	+25	145 (2)
19	*hsf-1(sy441)*	Control	11.5±1.9		14.4±0.8		287 (7)
20	*hsf-1(sy441)*	*acn-1*	13.0±2.1***	+12	16.1±0.8***	+11	413 (8)
21	*daf-16(mu86)*	Control	11.8±2.3		16.1±1.4		341 (5)
22	*daf-16(mu86)*	*acn-1*	11.4±2.5*	-3	15.8±1.3^ns^	-2	375 (5)
23	*daf-2(e1370)*	Control	39.3±8.3		52.7±1.6		208 (3)
24	*daf-2(e1370)*	*acn-1*	43.4±8.5***	+11	57.3±1.6***	+9	234 (3)
25	*age-1*(*hx546*)	Control	20.7±6.4		30.3±1.5		65 (2)
26	*age-1*(*hx546*)	*acn-1*	26.8±5.6***	+30	36.2±2.1***	+19	111 (2)
27	*age-1*(*am88*)	Control	22.9±6.2		33.6±1.5		95 (2)
28	*age-1*(*am88)*	*acn-1*	26.4±7.0***	+15	39.0±1.8***	+16	139 (2)

^1^Genotype: Wild-type hermaphrodites or the indicated mutants were analyzed.

^2^RNAi: Animals were cultured with bacteria containing the control RNAi plasmid (L4440) or the *acn-1* RNAi plasmid. Culture temperature was 20°C, except those labeled 25°C. Animals were cultured with RNAi bacteria starting at the embryonic stage (unlabeled) or the L4 stage (labeled adult).

^3^Mean, Maximum, % Change: Maximum adult lifespan is the mean lifespan of the 10% of the population that had the longest lifespans. Comparisons are to the paired control RNAi

*, *P* < 0.05

**, *P* < 0.005

***, *P* < 0.0001

n.s., not significant, P>0.05.

^4^N(n): Total number of hermaphrodites analyzed, and the number of independent experiments.

Several mutations have been identified that increase the sensitivity of worms to feeding RNAi, including mutation of *rrf-3* [38]. Feeding *acn-1* RNAi bacteria to *rrf-3* mutant animals beginning at the embryonic stage caused a significant increase of mean and maximum lifespan of 33% and 24%, respectively (Fig 2C, Table 2, line 5–6). Similarly, feeding *acn-1* RNAi beginning at the L4 stage caused a significant extension of mean and maximum lifespan of 46% and 33%, respectively (Fig 2D, Table 2, line 7–8). The extensions caused by *acn-1* RNAi in the *rrf-3* background were greater than the extensions in the wild-type background, indicating that *rrf-3* mutant animals are indeed more susceptible to the effect of the RNAi treatment. Moreover, *acn-1* RNAi also caused a significant extension of mean and maximum lifespan of *rrf-3* mutant animals at 25°C (S1B Fig, Table 2, line 9–10). To quantify how *acn-1* mRNA levels are affected by feeding RNAi, we performed quantitative RT-PCR. *acn-1* RNAi reduced mRNA levels about 50% compared to control RNAi in *rrf-3* mutant animals (S4 Fig).

### Reducing the activity of *acn-1* delayed age-related degenerative changes

**Fig 3:**
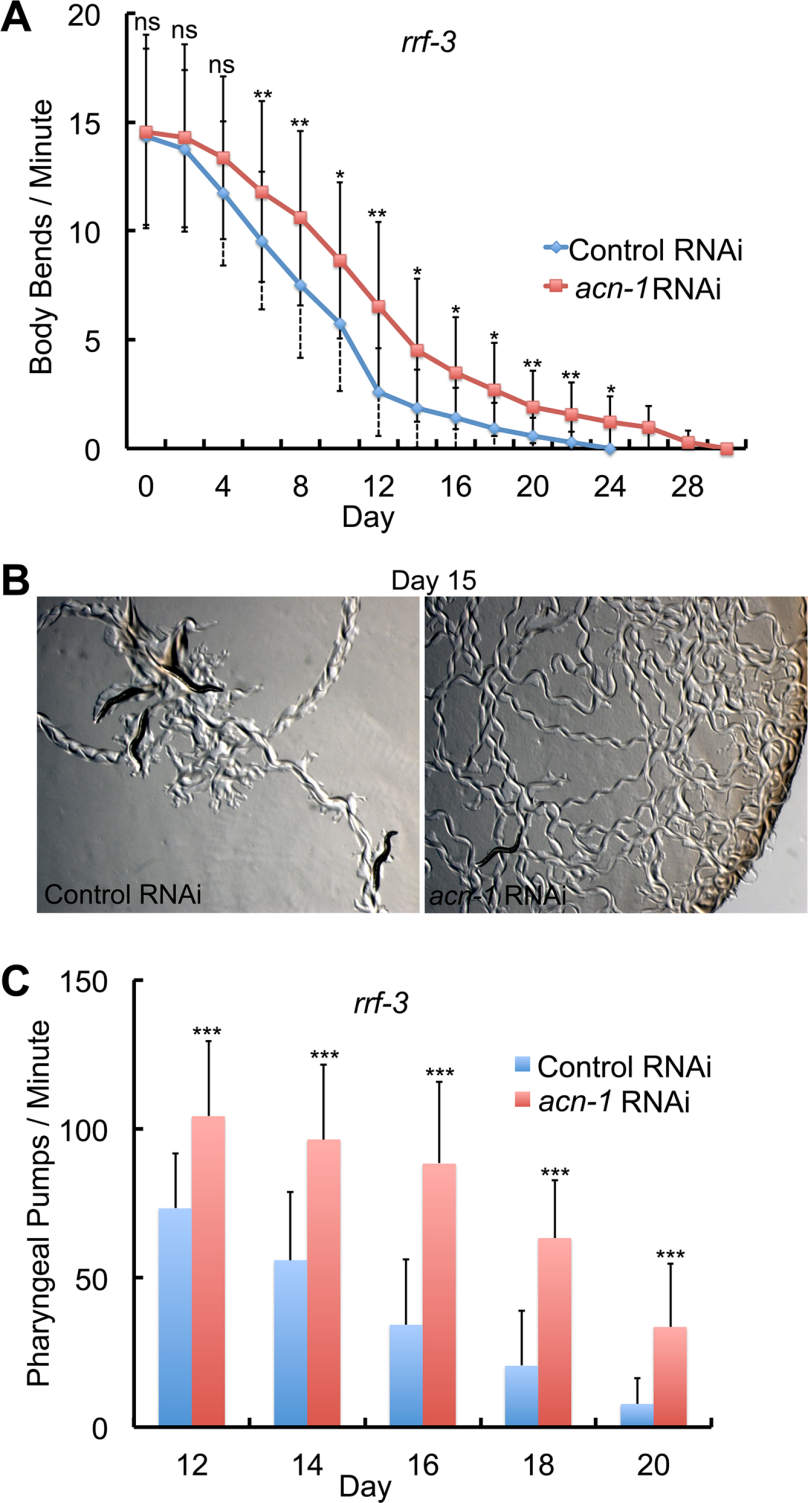
*acn-1* RNAi delayed age-related degenerative changes. *rrf-3(pk1426)* mutant hermaphrodites were cultured at 20°C with bacteria containing the control RNAi plasmid (L4440, blue) or the *acn-1* RNAi plasmid (red) starting at the embryonic stage. (A) Body movement was assessed by counting body bends with a dissecting microscope. (B) Images show bacterial lawns. Day 15 adults were cultured on fresh, smooth lawns for two hours–lines are tracks caused by moving worms. Animals treated with *acn-1* RNAi generated more tracks than control animals, and the tracks are suggestive of more coordinated sinusoidal movement compared to control animals. (C) Pharyngeal pumping was assessed by counting pumps with a dissecting microscope. n.s., *P* > 0.05; *, *P* < 0.05; **, *P* < 0.005; ***, *P* < 0.0001.

To characterize how *acn-1* influences age-related degeneration, we monitored age-related declines of major physiological processes. Wild-type *C*. *elegans* hermaphrodites display coordinated, sinusoidal body movement as young adults, and the frequency and coordination of body movement display age-related declines. To analyze body movement quantitatively, we counted body bends on solid NGM using a dissecting microscope. Hermaphrodites cultured with *acn-1* RNAi displayed a significantly higher rate of body movement beginning on day 4 of adulthood and extending to day 26 of adulthood (Fig 3A). To illustrate this difference, we exploited the fact that worms leave tracks in the bacterial lawn as they move. Five animals on day 15 of adulthood were transferred to fresh bacterial lawns, allowed to move for two hours, and the lawns were photographed. Fig 3B shows that hermaphrodites treated with control RNAi left a small number of tracks, and the tracks are suggestive of uncoordinated movement. By contrast, hermaphrodites treated with *acn-1* RNAi left abundant tracks that were suggestive of coordinated sinusoidal movement.

We monitored the age-related decline in pharyngeal pumping rate quantitatively by direct observation using a dissecting microscope. Hermaphrodites treated with *acn-1* RNAi displayed higher rates of pharyngeal pumping on days 12–20 of adulthood (Fig 3C). These results demonstrate that *acn-1* is necessary to promote the rapid, age-related decline of body movement and pharyngeal pumping observed in wild-type animals.

To analyze the effect on reproduction, we monitored progeny production of self-fertile hermaphrodites. Wild-type animals treated with *acn-1* RNAi did not display significant changes in total self fertile brood size or the daily production of progeny (S2C and S2D Fig). Captopril treatment only slightly delayed age-related changes of pharyngeal pumping, whereas *acn-1* RNAi significantly delayed age-related changes of pharyngeal pumping and body movement, suggesting *acn-1* RNAi may reduce the activity of *acn-1* to a greater extent than captopril or the drug may have toxic effects.

### Reducing the activity of *acn-1* increased stress resistance

**Fig 4:**
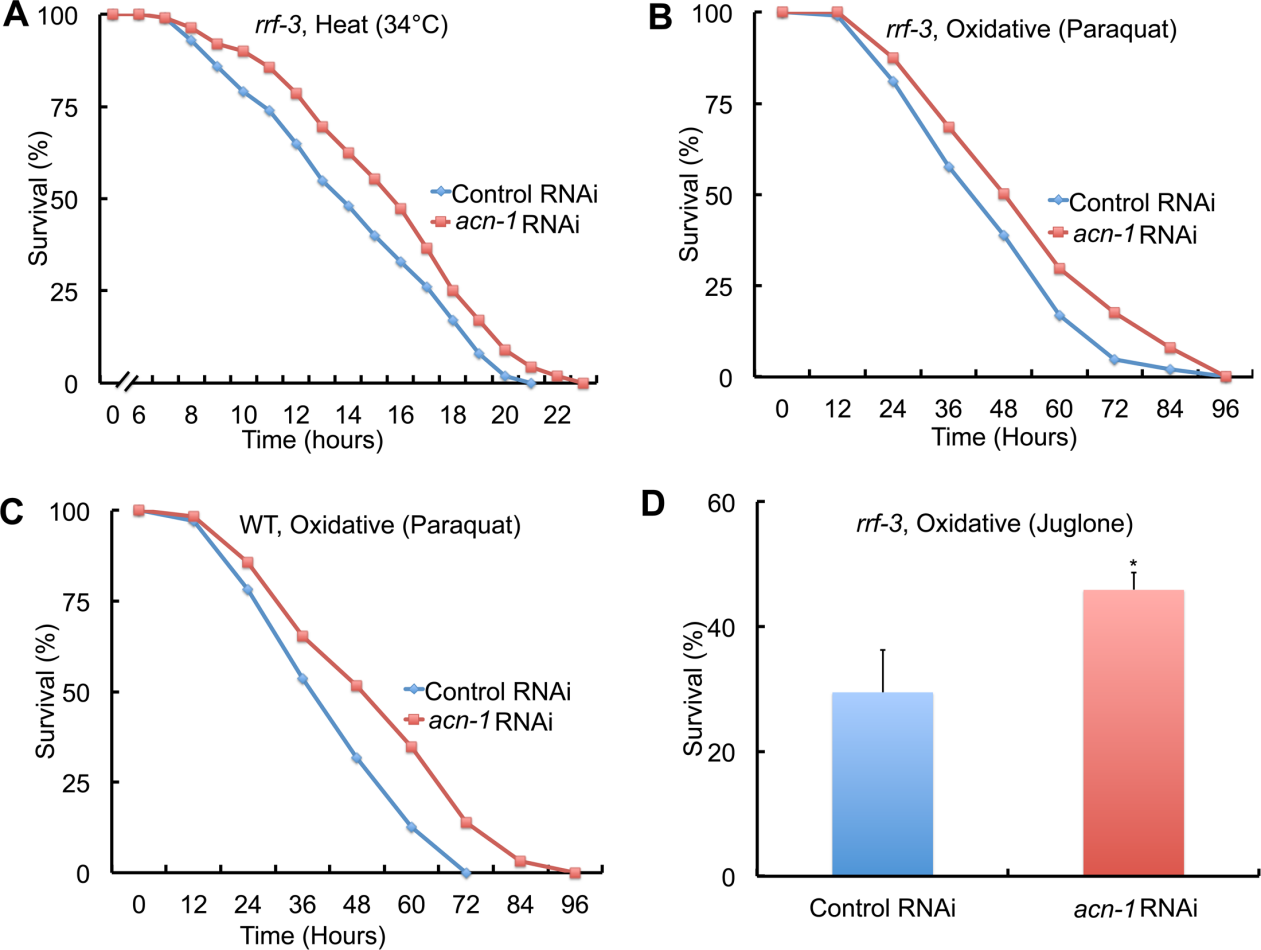
*acn-1* RNAi increased stress resistance. Hermaphrodites were cultured at 20°C with bacteria containing the control RNAi plasmid (L4440, blue) or the *acn-1* RNAi plasmid (red) starting at the embryonic stage. (A) *rrf-3* mutant animals were shifted to 34°C, a heat stress, and scored hourly for survival starting at 6 hours. *rrf-3* mutant (B) or WT (C) animals were transferred to NGM dishes with 40 mM paraquat, an oxidative stress, and scored every 12 hours for survival. (D) *rrf-3* mutant animals were transferred to liquid medium containing 240 uM juglone, another oxidative stress, and scored for survival after 9 hours. Bars indicate percent survival and standard deviation. See Table 3 for summary statistics, number of animals and number of independent experiments. *, *P* < 0.05.

Several *C*. *elegans* mutations that extend longevity also increase stress resistance [39,40]. To investigate the function of *acn-1* in stress resistance, we analyzed heat and oxidative stress. Embryos were cultured at 20°C with control RNAi or *acn-1* RNAi, and after 3 days animals were transferred to stressful conditions and monitored for survival. When exposed to continuous 34°C heat stress, control animals displayed a time dependent decrease in survival with a mean lifespan of 14.0 hours; animals treated with *acn-1* RNAi displayed a significant, 12% extension of mean lifespan of 15.7 hours (Fig 4A, Table 3, line 1–2). When exposed to oxidative stress caused by 40mM paraquat, control animals displayed a time dependent decrease in survival with a mean lifespan of 47.8 hours; animals treated with *acn-1* RNAi displayed a significant, 16% extension of mean lifespan of 55.4 hours (Fig 4B, Table 3, line 3–4). In addition, we observed a similar result of extended survival in oxidative stress when wild-type animals were treated with *acn-1* RNAi (Fig 4C Table 3, line 5–6). To determine if the specific conditions or oxidation generating chemical are important for the results, we analyzed oxidative stress in liquid medium using the compound juglone to cause oxidative stress. After nine hours of juglone exposure, animals treated with *acn-1* RNAi displayed a significant, 56% increase in survival compared to control animals (Fig 4D Table 3, line 7–8). These results indicate that the *acn-1* gene is necessary to promote wild-type levels of sensitivity to multiple stresses including heat and oxidation.

**Table 3:** *acn-1* RNAi caused resistance to oxidative and heat stress.

Line	Genotype^1^	RNAi^2^	Stress^3^	Mean^4^ lifespan ± SD (hours)	% Alive^4^ 9hrs	% Change^4^	N (n)^5^
1	*rrf-3(pk1426)*	Control	Heat Stress (34°C)	14.0±3.7	N/A		100 (3)
2	*rrf-3(pk1426)*	*acn-1*		15.7±3.7*		+12	112 (3)
3	*rrf-3(pk1426))*	Control	Oxidative stress (paraquat)	47.8±1.5	N/A		86 (3)
4	*rrf-3(pk1426)*	*acn-1*		55.4±1.7*		+16	73 (3)
5	WT	Control	Oxidative stress (paraquat)	44.8± 1.3	N/A		32 (2)
6	WT	*acn-1*		54.3±1.7*		+21	52(2)
7	*rrf-3(pk1426)*	Control	Oxidative stress (juglone)	N/A	29.5±6.8		134 (3)
8	*rrf-3(pk1426)*	*acn-1*			45.9±2.8*	+56	133 (3)

^1^Genotype: Wild-type hermaphrodites or *rrf-3(pk1426)* mutants were analyzed.

^2^RNAi: Animals were cultured with bacteria containing the control RNAi plasmid (L4440) or the *acn-1* RNAi plasmid starting at the embryonic stage. Culture temperature was 20°C, except during the heat stress.

^3^Stress: Animals were transferred to stressful conditions after 3 days, either 34°C heat stress, NGM dishes containing 40mM paraquat, or liquid medium containing 240uM juglone.

^4^Mean, % Alive, % Change: Mean lifespan was calculated using Excel. Comparisons are to the paired control RNAi

*, *P* < 0.05.

^5^N(n): Total number of hermaphrodites analyzed, and the number of independent experiments.

### Captopril and *acn-1* RNAi displayed similar interactions with mutations that influence lifespan

**Fig 5:**
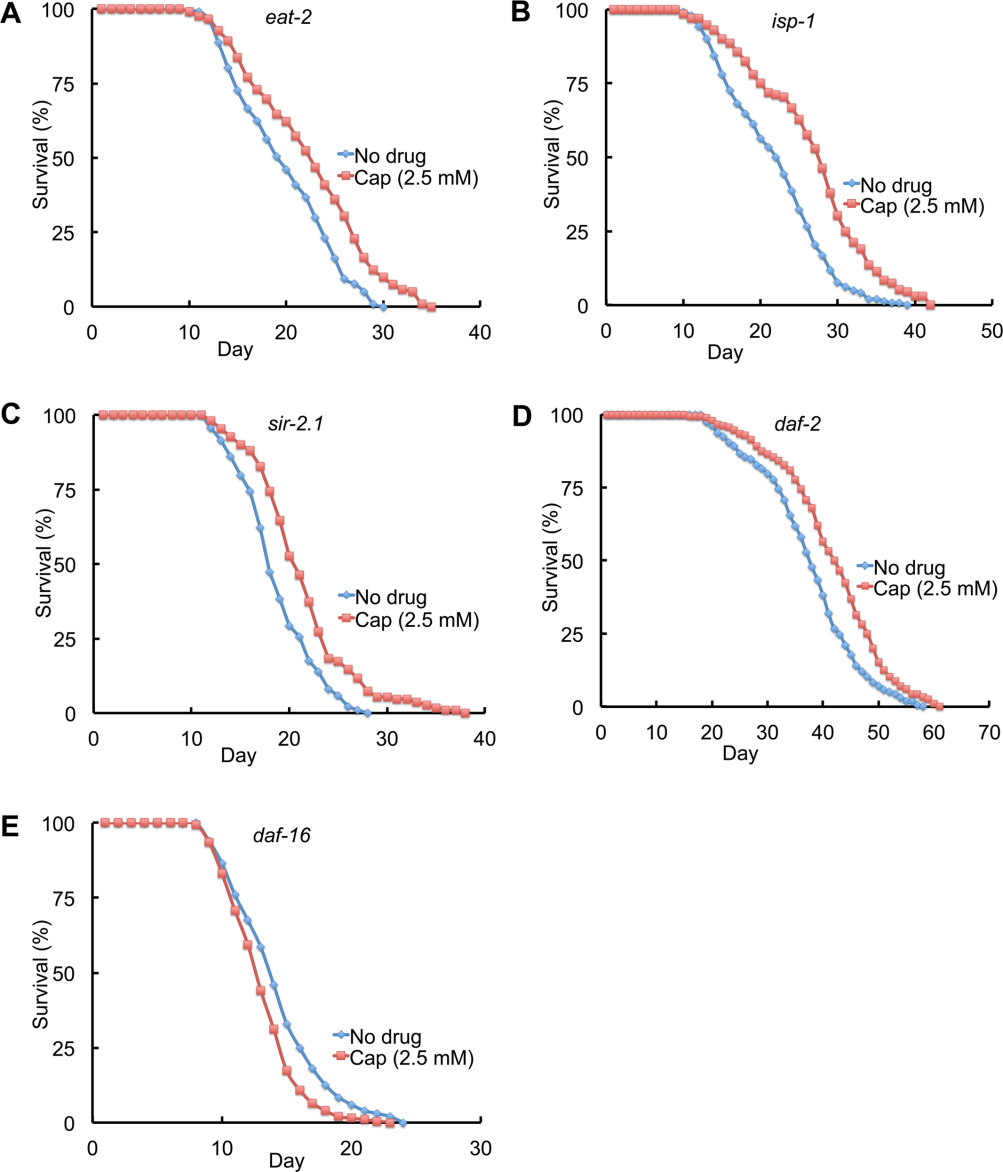
Captopril interactions with longevity pathways. Survival curves of mutant hermaphrodites cultured at 20°C with no drug or 2.54 mM captopril (Cap). Hermaphrodites were exposed to captopril starting at the L4 stage (day 0) and monitored regularly until death. Genotypes were (A) *eat-2(ad1116)*, (B) *isp-1(qm150)*, (C) *sir-2*.*1(ok434)*, (D) *daf-2(e1370)* and (E) *daf-16(mu86)*. See Table 1 for summary statistics, number of animals and number of independent experiments.

**Fig 6:**
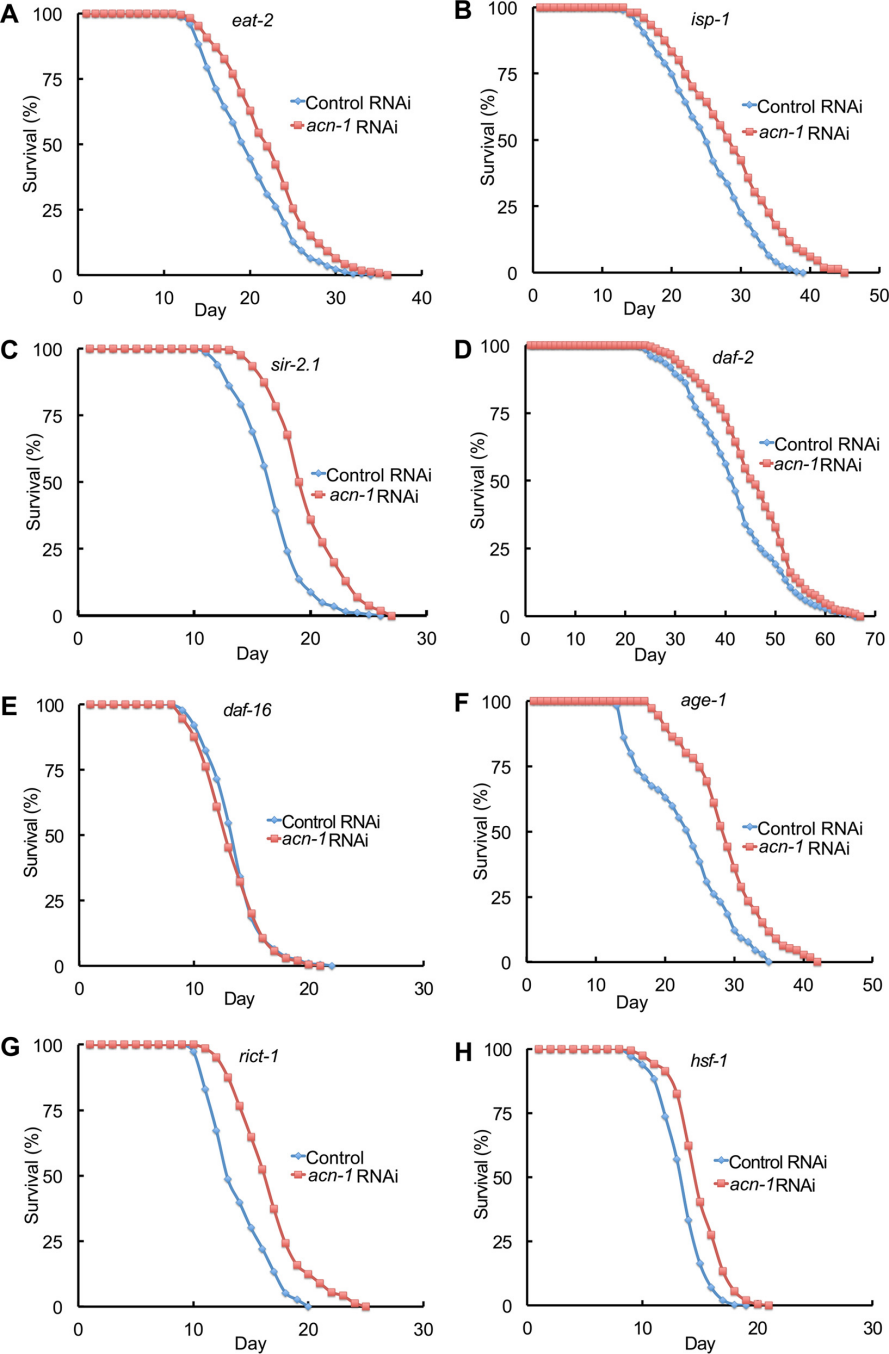
*acn-1* RNAi interactions with longevity pathways. Survival curves of mutant hermaphrodites cultured at 20°C with bacteria containing the control RNAi plasmid (L4440, blue) or the *acn-1* RNAi plasmid (red). Hermaphrodites were exposed to RNAi bacteria starting at the embryonic stage and monitored regularly until death. Genotypes were (A) *eat-2 (ad1116)*, (B) *isp-1 (qm150)*, (C) *sir-2*.*1 (ok434)*, (D) *daf-2 (e1370)*, (E) *daf-16 (mu86)*, (F) *age-1 (hx546)*, (G) *rict-1 (mg360)* and (H) *hsf-1 (sy441)*. See Table 2 for summary statistics, number of animals and number of independent experiments.

To investigate the mechanism of action of captopril and *acn-1* in lifespan extension, we analyzed how captopril treatment and *acn-1* RNAi affects animals with mutations that alter longevity. Caloric restriction extends the lifespan of many organisms, indicating that *ad libitum* feeding during laboratory culture reduces longevity. Mutations of the *eat-2* gene impair pharyngeal pumping, reduce food intake and cause a lifespan extension [16,41]. Captopril significantly extended the mean and maximum lifespan of *eat-2(ad1116)* mutant animals by 14% and 17%, respectively (Fig 5A; Table 1, line 13–14). Similarly, *acn-1* RNAi significantly extended mean and maximum lifespan by 12% and 10%, respectively (Fig 6A, Table 2, line 11–12). Thus, the lifespan extension caused by caloric restriction was additive with captopril treatment and *acn-1* RNAi.

Mutations of several genes that are important for mitochondrial function cause a lifespan extension in *C*. *elegans*, indicating that normal mitochondrial function promotes a rapid lifespan. The *isp-1* gene encodes a iron sulfur cluster containing protein that is important for the function of complex III to catalyze electron transport from ubiquinol to cytochrome c, and *isp-1* mutations extend lifespan [17,42]. Captopril treatment significantly extended the mean and maximum lifespan of *isp-1(qm150)* mutant animals by 23% and 20%, respectively (Fig 5B; Table 1, line 15–16). Similarly, *acn-1* RNAi significantly extended mean and maximum lifespan by 14% and 13%, respectively (Fig 6B, Table 2, line 13–14). Thus, the lifespan extension caused by reducing mitochondrial function was additive with captopril treatment and *acn-1* RNAi.

Overexpression of SIR2 (silent information regulator 2) is reported to extend the lifespan of several organisms, although this effect is not always observed [43,44]. In *C*. *elegans*, *sir-2*.*1* is predicted to encode a nicotinamide adenine dinucleotide (NAD) dependent deacetylase that can extend the lifespan of *C*. *elegans* when overexpressed. We examined the null mutation *sir-2*.*1(ok434)* [45]. Captopril treatment significantly extended the mean and maximum lifespan of *sir-2*.*1(ok434)* mutant animals by 17% and 27%, respectively (Fig 5C; Table 1, line 17–18). Similarly, *acn-1* RNAi significantly extended the mean and maximum lifespan of *sir-2*.*1* mutants by 16% and 18%, respectively (Fig 6C, Table 2, line 15–16). Thus, *sir-2*.*1* activity was not necessary for the lifespan extension activity of captopril or *acn-1* RNAi.

The target of rapamycin (TOR) signaling network plays an important role in nutrient homeostasis and influences adult lifespan [46]. Loss-of-function mutations in *rict-1* affect TOR signaling and cause a shorter lifespan. *acn-1* RNAi significantly extended the mean and maximum lifespan of *rict-1* mutants by 23% and 25%, respectively (Fig 6G, Table 2, line 17–18). *hsf-1* encodes a transcription factor that is important for stress response; overexpression of *hsf-1* extends lifespan and delays age-related protein miss folding [47], whereas reducing the activity of *hsf-1* causes a shorter lifespan and proteotoxicity [31,48,49]. *acn-1* RNAi significantly extended the mean and maximum lifespan of *hsf-1(lf)* mutants by 12% and 11%, respectively (Fig 6H, Table 2, line 19–20). Thus, the activities of *rict-1 and hsf-1* were not necessary for the lifespan extension caused by *acn-1* RNAi.

Mutations in the insulin/insulin-like growth factor (IGF) signaling pathway influence *C*. *elegans* lifespan [7,8,50–53]. Mutations that partially reduce the activity of *daf-2*, which encodes a protein homologous to the vertebrate insulin/IGF-1 receptor, or *age-1*, which encodes a protein homologous to the vertebrate PI3 kinase, extend lifespan. This signaling pathway controls the activity of a FOXO transcription factor encoded by *daf-16*, and *daf-16* activity is necessary for the lifespan extension caused by mutations in upstream signaling genes [13,14]. Thus, *daf-2* and *age-1* activity promote a rapid lifespan and inhibit longevity, whereas *daf-16* activity promotes longevity. Captopril treatment significantly extended the mean and maximum lifespan of *daf-2(e1370)* partial loss-of-function mutant animals by 11% and 9%, respectively (Fig 5D; Table 1, line 21–22). Similarly, *acn-1* RNAi significantly extended mean and maximum lifespan by 11% and 9%, respectively (Fig 6D, Table 2, line 23–24). In combination with an *age-1(hx546)* partial loss-of-function mutation that causes an extended lifespan, *acn-1* RNAi significantly extended mean and maximum lifespan by 30% and 19%, respectively (Fig 6F, Table 2, line 25–26). A similar result was obtained by analyzing *age-1(am88)* mutant animals (Table 2, line 27–28, S5 Fig) [54]. Thus, the lifespan extension caused by reducing *daf-2* activity was additive with captopril treatment and *acn-1* RNAi, and the lifespan extension caused by reducing *age-1* activity was additive with *acn-1* RNAi.

By contrast, captopril treatment did not extend the lifespan of *daf-16 (mu86)* loss-of-function mutant animals, but rather significantly shortened the mean and maximum lifespan by 10% and 11%, respectively (Fig 5E; Table 1, line 19–20). Similarly, *acn-1* RNAi slightly shortened the mean and maximum lifespan by 3% and 2%, respectively (Fig 6E, Table 2, line 21–22). These findings indicate that the lifespan extension activity of captopril and *acn-1* RNAi require *daf-16* activity; however, the reduction of lifespan raises the possibility that the combination of captopril treatment or *acn-1* RNAi and the *daf-16* mutation causes toxicity.

To investigate the possibility that *acn-1* functions upstream of *daf-16*, we analyzed additional phenotypes associated with the insulin/IGF-1 pathway. Upstream signaling proteins such as DAF-2 control the activity of DAF-16; specifically, *daf-2(lf)* mutations that cause a lifespan extension also cause DAF-16 protein to localize to the nucleus, where DAF-16 controls the activity of target genes [55]. To examine the nuclear localization of DAF-16, we used transgenic worms containing a DAF-16::GFP reporter construct [56]. Animals treated with captopril or *acn-1* RNAi did not display a substantial nuclear localization of DAF-16::GFP compared to control animals (S6C Fig, S1 Table). Thus, *acn-1* RNAi did not cause the same effect as a *daf-2(lf)* mutation, and *acn-1* is not necessary to inhibit nuclear localization of DAF-16. It has been proposed that *daf-16* is regulated by additional mechanisms that do not involve changes in subcellular localization, such as transcript levels [57], EAK-7 [58], and phosphorylation [59,60]. Our results do not exclude the possibility that captopril or *acn-1* RNAi regulate *daf-16* in a manner that does not change nuclear localization.

*daf-2(lf)* mutations cause a dauer constitutive (Daf-c) phenotype, indicating that *daf-2* is necessary to inhibit dauer development. To analyze the role of *acn-1* in dauer formation, we cultured worms with *acn-1* RNAi bacteria at 20°C, shifted embryos to 27°C to stimulate dauer formation, and scored dauer larvae after 72 hours. *acn-1* RNAi did not increase the frequency of dauer formation compared to control RNAi in wild-type animals or *rrf-3* mutant animals (S6A Fig). To increase the sensitivity of the assay, we analyzed the function of *acn-1* in *daf-2(lf)* mutants that display a partially penetrant, temperature sensitive Daf-c phenotype [61]. *acn-1* RNAi was not different from control RNAi in this assay (S6B Fig). Thus, *acn-1* RNAi did not cause the same effect on dauer formation as a *daf-2(lf)* mutation, and *acn-1* is not necessary to inhibit formation of dauer larvae.

### The lifespan extensions caused by captopril and *acn-1* RNAi were not additive

**Fig 7:**
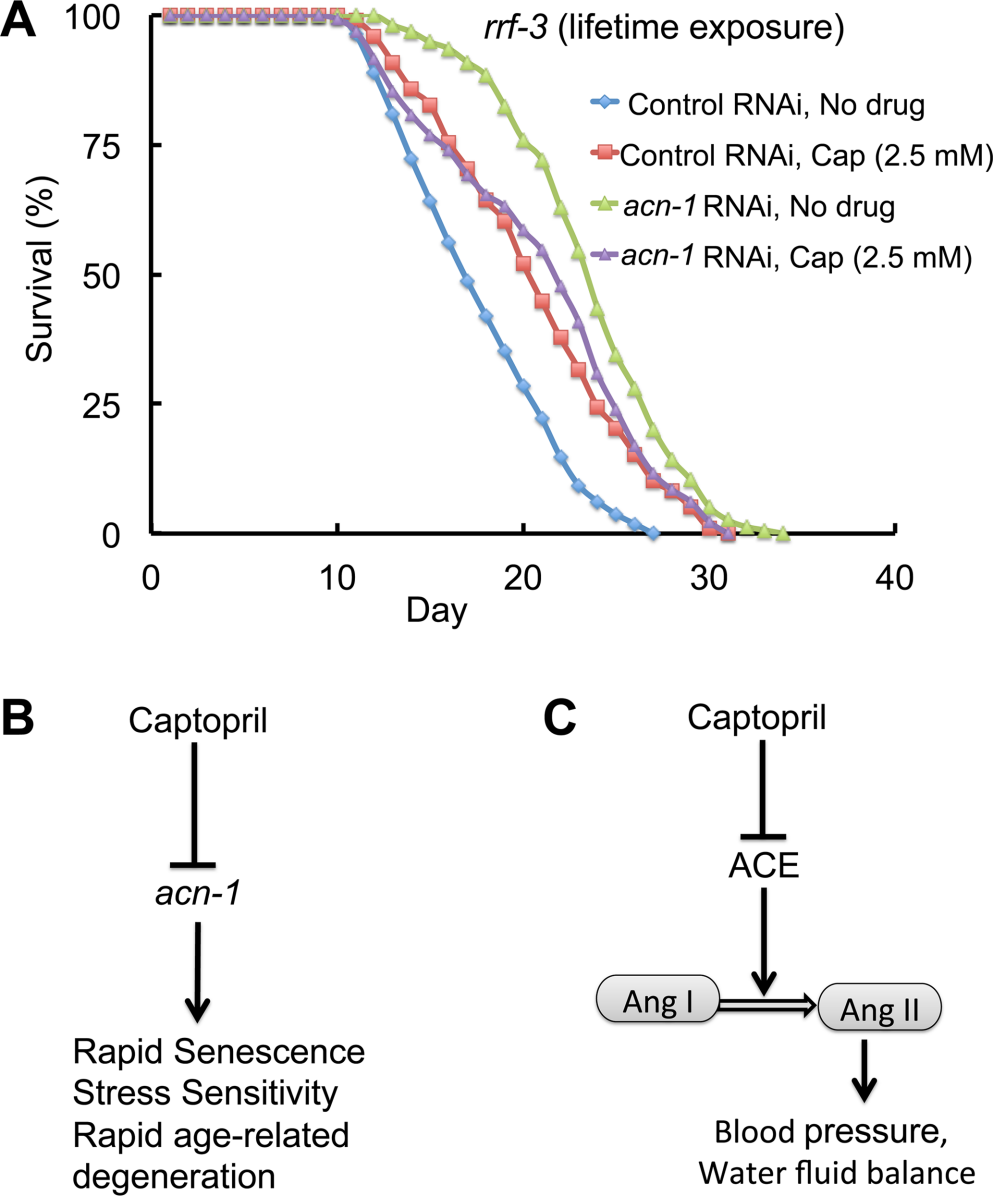
Captopril extends lifespan by reducing the activity of *acn-1*. (A) Survival curves of *rrf-3(pk1426)* mutant hermaphrodites cultured at 20°C with bacteria containing the control RNAi plasmid (L4440) or the *acn-1* RNAi plasmid. Hermaphrodites were exposed to RNAi bacteria starting at the embryonic stage. Hermaphrodites were cultured with no drug or 2.54 mM captopril (Cap) starting at the L4 stage (day 0). See Table 4 for summary statistics, number of animals and number of independent experiments. (B) A model illustrating the relationship between captopril and *acn-1* in *C*. *elegans*. Bars indicate a negative effect, and arrows indicate a positive effect. (C) In humans, captopril inhibits angiotensin-converting enzyme (ACE), which catalyzes the cleavage of angiotensin I to angiotensin II. Angiotensin II promotes high blood pressure and has other effects on physiology [26,27].

We hypothesized that captopril inhibits *acn-1* to extend lifespan. This hypothesis predicts that the effects of captopril and *acn-1* RNAi will not be additive, because our dose-response analysis indicates that we have identified the optimal dose of captopril for lifespan extension. To test this prediction, we combined treatment with captopril and *acn-1* RNAi. Captopril treatment alone caused a 20% extension of mean lifespan to 18.7 days, whereas *acn-1* RNAi alone caused a 38% extension of mean lifespan to 21.5 days (Fig 7A, Table 4, line 1–3). Combining captopril treatment and *acn-1* RNAi resulted in a 18.9 day lifespan that was not significantly different from captopril treatment alone and significantly shorter than *acn-1* RNAi treatment alone. Thus, captopril and *acn-1* RNAi did not have an additive effect on lifespan extension, consistent with the model that both effects are mediated by a similar mechanism.

**Table 4:** Captopril and *acn-1* RNAi were not additive.

Line	Genotype^1^	RNAi^2^	Drug^3^	Mean^4^ Lifespan ± SD (days)	%^4^ Change	Maximum^4^ Lifespan ± SD (days)	% ^4^ Change	N (n)^5^
1	*rrf-3(pk1426)*	Control	None	15.6±4.1		23.1±1.2		162 (3)
2	*rrf-3(pk1426)*	Control	Captopril (2.54 mM)	18.7±5.0**	+20	27.2±0.9***	+18	98 (3)
3	*rrf-3(pk1426)*	*acn-1*	None	21.5±4.4***	+38	28.7±1.2***	+24	154 (3
4	*rrf-3(pk1426)*	*acn-1*	Captopril (2.54 mM)	18.9±5.6	+1/-12	27.5±1.0	+1/-4	130 (3)

^1^Genotype: *rrf-3(pk1426)* hermaphrodites were analyzed.

^2^RNAi: Animals were cultured at 20°C with bacteria containing the control RNAi plasmid (L4440) or the *acn-1* RNAi plasmid starting at the embryonic stage.

^3^Drug: Animals were cultured on standard NGM (None) or with NGM containing 2.54 mM captopril starting at the L4 stage.

^4^Mean, Maximum, % Change: Maximum adult lifespan is the mean lifespan of the 10% of the population that had the longest lifespans. Lines 2 and 3 are compared to line 1, and line 4 is compared to line 2/line 3. There was no significant difference between lines 4 and 2, whereas line 4 was significantly shorter then line 3 (*P* < 0.05 or *P* < 0.0001).

*, *P* < 0.05

**, *P* < 0.005

***, *P* < 0.0001; n.s., not significant, P>0.05.

^5^N(n): Total number of hermaphrodites analyzed, and the number of independent experiments.

## Discussion

### Identification of captopril as a new drug that extends *C*. *elegans* longevity

The identification of compounds that can delay age-related degeneration and extend lifespan is an important goal of aging research, because age-related decline is a major cause of disability and death in humans, and so far no compounds have been demonstrated to delay human aging. We reasoned that FDA-approved drugs used to treat human diseases might also influence aging and lifespan. To identify such drugs, we screened examples of different structural and functional drug classes. We previously described the identification of anticonvulsant drugs such as ethosuximide and the neuroactive drug valproic acid [19,20]. Here we identified the blood pressure medicine captopril as a way to extend *C*. *elegans* lifespan. The effect of captopril was dose dependent; at an optimal dose, captopril significantly extended mean lifespan 22–28% and maximum lifespan 18–32%. Captopril extended lifespan at a variety of temperatures and in a variety of mutant backgrounds, indicating that the effect is robust in the face of environmental and genetic variation. Captopril functioned in adult animals to extend lifespan, suggesting that it affects the rate of age-related decline rather than developmental processes.

The first of what is now a large class of ACE inhibitors, captopril is an oligopeptide derivative developed in 1975 based on a peptide found in pit viper venom [62]. ACE inhibitors modulate the renin-angiotensin-aldosterone system, a mechanism by which the body adapts to hypotension [63]. In response to a decline in blood pressure, the kidney releases renin, which cleaves angiotensinogen to angiotensin I. ACE converts angiotensin I to angiotensin II, and angiotensin II acts through a transmembrane receptor to stimulate aldosterone secretion and promote vasoconstriction to increase blood pressure. By blocking ACE and preventing the conversion of angiotensin I to angiotensin II, captopril lowers blood pressure.

Two strategies have been used to identify compounds that can extend *C*. *elegans* lifespan: screening chemical libraries and testing candidate compounds based on the hypothesis that the target of the drug may influence aging and longevity [64]. Compounds that have been tested included FDA-approved drugs, libraries of chemically defined molecules, and extracts of plants that contain a mixture of chemicals. Library screening resulted in the identification of antidepressant drugs [21,25]. Candidate compounds that have been reported to extend worm lifespan include resveratrol [65], trehalose [24], lithium [66] and garlic constituent [67]. Extracts of blueberries and ginkgo have been reported to extend worm lifespan [68,69]. ACE inhibitors such as captopril have not been previously reported to extend lifespan in worms, so our findings identify a new chemical entity that influences aging in *C*. *elegans*.

### Captopril inhibits *acn-1* to delay aging in *C*. *elegans*

It is well established that ACE is the target that mediates the effect of captopril on blood pressure in humans [62]. ACE genes have been highly conserved during evolution, and *acn-1* encodes the *C*. *elegans* homolog of ACE [28]. A major issue in aging pharmacology is the identification of the direct target of the drug, and in most cases the targets of drugs that extend *C*. *elegans* lifespan remain unknown. We hypothesized that captopril inhibits ACN-1 to extend longevity. This hypothesis makes three important predictions that were verified experimentally. First, it predicts that reducing the activity of *acn-1* using genetic techniques can extend longevity. We showed that targeting *acn-1* by RNAi increased mean lifespan 20–46% and maximum lifespan 18–33%. Second, it predicts that reducing the activity of *acn-1* and treatment with captopril will cause similar effects in a variety of genetic backgrounds. Indeed, captopril treatment and reducing *acn-1* activity gave very similar results in five genetic backgrounds (*eat-2*, *isp-1*, *sir-2*.*1*, *daf-16* and *daf-2*) and at two temperatures. In addition, both treatments function in adults to extend longevity. Third, it predicts that the lifespan extension caused by captopril treatment and reducing *acn-1* activity will not be additive. This prediction was also verified. While these results are consistent with captopril inhibition of ACN-1, they do not demonstrate that the drug directly binds ACN-1 protein or inhibits a biochemical activity of ACN-1. The biochemical activity of ACN-1 has not been established, and ACN-1 may not have protease activity because critical residues in the predicted active site have not been conserved during evolution [28]. Further studies are necessary to establish an assay for the biochemical activity of ACN-1 and directly test the effect of captopril.

The expression and function of *acn-1* were analyzed by Brooks *et al*. [28] using a reporter gene encoding ACN-1::GFP and *acn-1* RNAi, respectively. *acn-1* is expressed in embryonic and larval hypodermis, in the vulva during organogenesis and in the ray papillae of the male tail. RNAi delivered by injection in the gonad caused larvae to arrest at the L2 stage and display evidence of molting defects. RNAi delivered by feeding to L1/L2 larvae caused a cuticle defective phenotype in L3/L4 larvae and adults. The failure to shed cuticle led to secondary defects such as vulva defects and constipation. These results indicate *acn-1* is necessary for larval molting, mail tail development and formation of adult alae. Frand *et al*. [70] identified *acn-1* in a genome-wide feeding RNAi screen for molting defects. An ACN-1::GFP transgene was expressed in the hypodermis, including the major body syncytium, hyp7, and hypodermal cells in the head and tail, the lateral seam cells, and the excretory gland cell. Neither of these studies describe aging phenotypes, so our results establish a new phenotype for *acn-1* and a novel link between *acn-1* and aging. The previously reported molting defects caused by *acn-1* RNAi are partially penetrant [28,70]; we did not observe a significant penetrance of molting defects, which may indicate less extreme gene disruption in our studies resulting from differences between the feeding RNAi constructs or the conditions of RNAi delivery.

To elucidate the role of captopril and *acn-1* in aging, we analyzed interactions with established pathways that influence longevity. Many mutations used in these experiments are not null alleles, and therefore the observation that the effects are additive does not exclude the possibility that two interventions act in the same pathway. Captopril treatment or reducing the activity of *acn-1* was additive with the lifespan extensions caused by an *eat-2* mutation that causes caloric restriction. Furthermore, these treatments did not reduce self-fertile brood size and reproductive span like caloric restriction, suggesting that captopril and *acn-1* do not act by causing caloric restriction. Captopril treatment or reducing the activity of *acn-1* was additive with the lifespan extensions caused an *isp-1* mutation that reduces mitochondrial activity, suggesting these treatments do not reduce mitochondrial function. The lifespan extension caused by reducing the activity of *acn-1* was not abrogated by loss-of-function mutations of *sir-2*.*1*, *hsf-1* or *rict-1*, suggesting that *acn-1* does not act by regulating these genes. Captopril treatment and reducing the activity of *acn-1* displayed complex interactions with the insulin/IGF-1 pathway. These treatments were additive with the lifespan extensions caused by loss-of-function mutations of *daf-2* and *age-1*. However, the lifespan extensions caused by both treatments were abrogated by a *daf-16* mutation. To further analyze the relationship with *daf-16*, we demonstrated that reducing the activity of *acn-1* did not cause dauer formation and did not promote nuclear localization of DAF-16, which are typical of reducing insulin/IGF-1 signaling upstream of *daf-16*. Thus, *acn-1* does not appear to act upstream and regulate the nuclear localization activity of *daf-16*. It is possible that *daf-16* is necessary because it functions in parallel to *acn-1* or that toxicity develops in the absence of both *daf-16* and *acn-1*. Overall, *acn-1* defines a new gene that influences longevity, and interactions with known longevity pathways suggest that it functions by a mechanism that is distinct from those that have been characterized previously.

### The ACE pathway may have a conserved function influencing aging in mammals

The ACE inhibitor enalapril and the angiotensin II receptor antagonist losartan have been reported to extend the life span of mice and rats [71–78]. Furthermore, these drugs delay the age-related degeneration of tissue structure and function in the kidney, cardiovascular system, liver and brain. Similarly, Santos *et al*., [79] showed that enalapril increased life span in rats. These interesting results indicate that the renin-angiotensin-aldosterone system promotes age-related degeneration, and blocking this system can extend longevity in rodents. The mechanism of these drugs in life span extension is not well defined–the affects are not well correlated with changes in blood pressure but may reflect preservation of mitochondrial number and function. Genetic studies reported by Benigni *et al*., [80] provide important support for these pharmacology studies, since disruption of the angiotensin II type I receptor (AT_1_) promotes longevity in mice. These results may be relevant to humans, since polymorphisms in the angiotensin II type I receptor gene are associated with extreme human longevity [81]. Overall, these studies suggest that the rennin-angiotensin-aldosterone system controls longevity in mammals. Thus, our discoveries in worms are likely to be relevant to mammalian biology. An important issue that has not been established by studies of mammals is the mechanism of action of this pathway in influencing aging and longevity. The results presented here provide new insights into the mechanism of action of captopril in lifespan extension and establish the powerful *C*. *elegans* system to investigate critical questions about the conserved activity of the pathway.

## Materials and Methods

### General methods and strains

*C*. *elegans* were cultured on 6 cm Petri dishes containing NGM agar and a lawn of *Escherichia coli* strain OP50 at 20°C unless stated otherwise [2]. The wild-type (WT) strain was N2 Bristol. *daf-2(e1370P1465S*) is a partial loss-of-function mutation that affects the kinase domain of the DAF-2 receptor tyrosine kinase [50]. *age-1(hx546P806S)* and *age-1(am88E725K)* are partial loss-of-function mutations that affect the AGE-1 PI3 kinase [53,54,82]. *daf-16(mu86*) is a strong loss-of-function mutation caused by a deletion in the DAF-16 forkhead transcription factor [13,14]; *eat-2(ad1116)* is a change in a splicing site predicted to decrease the level of mRNA of the EAT-2 non-alpha nicotinic acetylcholine receptor [41]; *isp-1(qm150P225S)* is a loss-of-function mutation that affects an iron sulfur protein of mitochondrial complex III [42]; *sir-2*.*1(ok434)* is a deletion that causes a loss-of-function of the SIR-2.1 NAD dependent protein deacetylase [45]. *rict-1*(*mg360G1067E*) is a partial loss-of-function mutation of RICT-1, a component of the target of rapamycin complex 2 (TORC2) that encodes an ortholog of mammalian Rictor [83]. *hsf-1*(*sy441W585stop*) is a strong loss-of-function mutation of the HSF-1 transcription factor [84]. DAF-16 nuclear localization was analyzed using strain GR1352 containing the integrated array *xrIs87* [DAF-16alpha::GFP::DAF-16B + *rol-6(su1006)]* [85]. The *rrf-3(pk1426)* mutation was used for RNAi feeding experiments [38].

### Screening for drugs that extend *C*. *elegans* lifespan

Fifteen FDA-approved drugs were screened for extension of *C*. *elegans* lifespan using methods described by Evason *et al*., [19] (atropine, yohimbine hydrochloride, captopril, nicotinic acid, phenformin, haloperidol, acetazolam, adenosine, cimetidine, lidocaine, procainamide hydrochloride, caramazepine, 5’-5’-diphenylhydation Sodium, caffeine and imipramine). For each drug, we analyzed about 50 hermaphrodites cultured with three concentrations in the NGM medium (X, 10-100X, 1000X). The lowest dose (X) was approximately equivalent to the effective dose in humans [63]. Captopril was obtained from Sigma Aldrich (St. Louis, MO, USA), and a 30 mg/ml stock solution was prepared by dissolving the compound in water. Concentrated captopril was diluted to the desired final concentration in liquid NGM that had been autoclaved and cooled to 55°C, and 7–8 ml of medium was dispensed into 6 cm Petri dishes. Petri dishes were allowed to dry 1–2 days at room temperature and then seeded with *E*. *coli* OP50. Lifespan experiments using dishes containing drugs were always conducted in parallel with control dishes containing no drug in the same incubator to control for day-to-day variations in temperature and humidity.

### Measurement of lifespan and age-related changes in physiological process

Studies of lifespan were begun on day zero by placing approximately 30–40 L4 hermaphrodites on a Petri dish. Each hermaphrodite was transferred to a fresh Petri dish daily during the reproductive period (approximately the first seven days) to eliminate self-progeny and every 2–3 days thereafter. Each hermaphrodite was examined every day using a dissecting microscope for survival, determined by spontaneous movement or movement in response to prodding with a pick. Dead worms that displayed matricidal hatching, vulval extrusion or desiccation due to crawling off the agar were excluded from the data analysis. Average mean lifespan was calculated as the number of days from the L4 stage to the last day a worm was observed to be alive. To conduct experiments with dead bacteria, we seeded dishes with live E. *coli* OP50, cultured for 24 hours, and exposed the bacteria to ultraviolet light by placing dishes in a UV Stratalinker 2400 for 15 minutes. Death was confirmed by inoculating LB medium with treated bacteria and observing no growth.

To analyze progeny production, one L4 hermaphrodite was placed on a Petri dish (day one), transferred to a fresh dish daily until at least 4 days without progeny production, and progeny were counted after two days. Pharyngeal pumping and body movement were determined as described previously [33]. Briefly, we observed pharyngeal pumping using a dissecting microscope for a 10 seconds interval. Body movement was assayed by observation using a dissecting microscope for 20 seconds. Petri-dishes were tapped to stimulate animals to move before scoring.

### RNA interference

RNAi interference was performed by feeding bacteria that express dsRNA as described by Kammath *et al*., [86]. Briefly, *E*. *coli* HT115 bacteria with the control plasmid (L4440) or a plasmid encoding *acn-1* were obtained from the Ahringer library [37], and the identity of the clone was confirmed by DNA sequencing. The *daf-2* RNAi bacterial strain was provided by M. Crowder. RNAi bacteria were streaked on LB dishes containing 50μg/ml ampicillin and 12.5 μg/ml tetracycline. Control and *acn-1* RNAi cultures were grown for 6 hours in LB medium containing 50μg/ml ampicillin. *Escherichia Coli* expressing double-stranded *acn-1* RNA did not form thick lawns on RNAi NGM agar dishes containing isopropyl β-d-1-thiogalactopyranoside (1 mM) and 50μg/ml carbenicillin, indicating double stranded *acn-1* RNA might inhibit bacterial proliferation. To address this issue, we prepared 3X-concentrated liquid bacterial culture from both control and *acn-1* RNAi bacteria, spread this on NGM RNAi dishes, and allowed dishes to incubate overnight. L4 stage larvae were transferred to RNAi dishes and cultured for one day, adults were transferred to a fresh RNAi dish and cultured for one day and then removed. Larva that developed on these plates were analyzed.

### Dauer formation and DAF-16::GFP nuclear localization

Dauer formation was assayed as described by Kimura *et al*., [50]. Briefly, we collected eggs from wild-type or *rrf-3(pk1426)* hermaphrodites cultured at 20°, transferred the eggs to 27°C with ample food, cultured for 72 hr, and examined hatched animals. Animals were classified as non-dauer (including adults and non-dauer larvae) or dauer on the basis of morphological criteria [61]. To analyze dauer formation of *daf-2(e1370)* mutant animals, we transferred eggs to 15°C, 17.5°C, 20°C, 22.5°C, or 25°C. For dauer formation experiments, we performed *acn-1* RNAi using the feeding protocol described by Kammath *et al*., [86]. Briefly, L4 stage hermaphrodites were transferred to dishes with control (L4440) or *acn-1* RNAi bacteria at 20°C, and embryos were transferred to fresh dishes with RNAi bacteria at the appropriate temperature and cultured for 3 or 4 days.

To analyze DAF-16::GFP localization, we used the strain GR1352 [85]. L4 stage animals were transferred to dishes seeded with control (L4440) and *acn-1* RNAi bacteria. Progeny were analyzed at the one day old adult stage using an Olympus SZX12 dissecting microscope (Tokyo, Japan) equipped for fluorescence microscopy. To reduce bias, the scoring was done by an observer blind to the RNAi treatment status. We analyzed each worm as having (1) GFP diffusely localized in the cytosol, (2) GFP localized in nuclei displaying intensely fluorescing puncta throughout the entire body from head to tail or (3) intermediate nuclear localization of GFP, defined as puncta observed in at least one or more nuclei but not in most or all nuclei. To perform the data analysis, we combined the nuclear and intermediate nuclear categories.

### Heat and oxidative stress assays

Thermotolerance assays were performed as described by McColl *et al*., [87]. Briefly, L4 stage hermaphrodites were cultured at 20°C on control (L4440) and *acn-1* RNAi dishes for 3 days. To perform the heat stress assay, we transferred adults to 34°C and scored the percentage of dead and live animals starting at 6 hours and continuing every hour until all animals died. Animals were scored as dead if they did not respond to a mechanical stimulus. To perform oxidative stress assays, we transferred day 3 adult hermaphrodites to NGM dishes containing 40 mM paraquat and scored for survival every 12 hours. For the heat stress and paraquat stress assays, animals that displayed matricidal hatching or vulval extrusion were not included in the data analysis. To perform oxidative stress assays with juglone, we transferred day 3 adult hermaphrodites to 2 ml of liquid M9 medium containing 240 uM juglone in an 18 well dish. Worms were scored for survival after 9 hours. Paraquat and juglone were obtained from Sigma Aldrich (St. Louis, MO, USA).

### Quantitative real-time PCR (RT–PCR)

To quantify mRNA levels, we cultured *rrf-3* adult worms on control and *acn-1* RNAi dishes for 3–4 hours to obtain synchronized eggs, removed adult worms, and continued culture until the eggs developed into two day old adult worms. These adults were washed and collected for RNA isolation. RNA analysis was performed as previously described with modifications [88]. Briefly, RNA was isolated using Trizol (Invitrogen) and treated with DNAse 1 enzyme. cDNA was synthesized by using High Capacity cDNA Reverse Transcription kit (Applied Biosystems). Quantitative, realtime PCR was performed using an Applied Biosystems Step One Plus Real-Time PCR system and iTaq Universal SYBR Green Supermix (BioRad Laboratories, Hercules, CA). mRNA fold change was determined by comparing *acn-1* mRNA levels with mRNA levels of the reference gene *rps-23*. Forward and reverse amplification primers were: *rps-23* 5′- aaggctcacattggaactcg and 5′- aggctgcttagcttcgacac; *acn-1* 5′- gtactacgagccactcatcaac and 5′- gaatctcctcgacagtgaatg.

### Statistical analysis

All data were analyzed using the two-tailed student t-test for samples with unequal variances by using Excel and http://studentsttest.com. P values less than 0.05 were considered statistically significant. To determine if the choice of a statistical test affected the conclusions, we used the log rank (Mantel-Cox) method to analyze a subset of the lifespan experiments. Both tests produced similar P values.

## Supporting Information

S1 Fig Captopril and *acn-1* RNAi extended adult lifespan at 25°C, and captopril extended adult lifespan when cultured with dead bacteria. **(A)** Survival curves of wild-type (WT) hermaphrodites cultured at 25°C with no drug or 2.54 mM captopril (Cap) in the NGM medium. Hermaphrodites were exposed to captopril starting at the L4 stage (day 0) and monitored regularly until death. See Table 1 for summary statistics, number of animals and number of independent experiments. **(B)** Survival curves of *rrf-3* mutant hermaphrodites cultured at 25°C with bacteria containing the control RNAi plasmid (L4440, blue) or the *acn-1* RNAi plasmid (red). Hermaphrodites were exposed to RNAi bacteria starting at the embryonic stage. See Table 2 for summary statistics. These data represent a single experiment (N = 50). **(C)** Survival curves of wild-type (WT) hermaphrodites cultured at 20°C with *E*. *coli* OP50 that was killed by exposure to ultraviolet light with no drug or 2.54 mM captopril (Cap) in the NGM medium. Hermaphrodites were exposed to captopril starting at the L4 stage (day 0) and monitored regularly until death. See Table 1 for summary statistics, number of animals and number of independent experiments.
(TIF)

S2 Fig Captopril did not strongly affect the age-related decline of pharyngeal pumping. Bars show the pharyngeal pumping rate in beats per minute and the standard deviation. The rate was measured by counting beats for 10 seconds using a dissecting microscope. Wild-type animals were treated with no drug (blue) or 2.54 mM captopril (red) starting at the L4 stage (day 0) and cultured at 20°C (N = 25). n.s., not significant, *P* > 0.05. Captopril treated animals displayed a small increase in pumping rate at days 5–9, but this trend was not statistically significant with this sample size.
(TIF)

S3 Fig Captopril and *acn-1* RNAi did not strongly affect self-fertile reproduction. Wild type self-fertile hermaphrodites were cultured at 20°C and treated with (A, B) no drug (blue) or 2.54 mM captopril (red) starting at the L4 stage (day 0). (C, D) Animals were cultured with RNAi bacteria containing the control RNAi plasmid (L4440, blue) or the *acn-1* RNAi plasmid (red). Hermaphrodites were exposed to RNAi bacteria starting at the embryonic stage. (A, C) Bars show total number of live progeny and standard deviation. (B, D) Data points show total number of live progeny produced each day. Number of animals analyzed: no drug (N = 3), captopril (N = 3), Control RNAi (N = 5) and *acn-1* RNAi (N = 5). n.s., not significant, *P* > 0.05.
(TIF)

S4 Fig Treatment with *acn-1* RNAi reduced the level of *acn-1* mRNA. mRNA was isolated from populations of two day old adult *rrf-3(pk1426)* animals cultured with control RNAi (blue) or *acn-1* RNAi (red). *acn-1* transcript levels were analyzed by RT-PCR; mRNA levels are expressed in arbitrary units (A.U.) and were normalized to *rps-23*, a ribosomal protein. The values were normalized by setting the value for control RNAi equal to 1.0. Bars represent the average +/- S.E.M. (n = 3 biological replicates).
(TIF)

S5 Fig *acn-1* RNAi extended the lifespan of *age-1(am88)* mutant animals. Survival curves of *age-1(am88)* mutant hermaphrodites cultured at 20°C with bacteria containing the control RNAi plasmid (L4440, blue) or the *acn-1* RNAi plasmid (red). Hermaphrodites were exposed to RNAi bacteria starting at the embryonic stage and monitored regularly until death. See Table 2 for summary statistics, number of animals and number of independent experiments.
(TIF)

S6 Fig *acn-1* RNAi did not cause a Daf-c phenotype or nuclear localization of DAF-16::GFP. Bars (A) and data points (B) indicate the percent of embryos that formed dauer larvae and standard deviation. Adult hermaphrodites were cultured at 20°C with bacteria containing the control RNAi plasmid (L4440, blue) or the *acn-1* RNAi plasmid (red). Embryos were cultured for three days at 27°C (A) or the indicated temperature (B) and scored for dauer larvae formation based on morphological criteria using a dissecting microscope. (A) Genotypes were wild type and *rrf-3(pk1426)*. (B) *daf-2* (*e1370*) caused a temperature sensitive Daf-c phenotype when cultured with no RNAi (*E*. *coli* OP50 bacteria) (purple triangles). Control RNAi and *acn-1* RNAi both increased the penetrance of the Daf-c phenotype to a similar extent, indicating that the effect is caused by the bacterial strain used for RNAi rather than the inhibition of the *acn-1* gene. Comparisons are to the paired control RNAi: n.s., not significant, *P* > 0.05. (C) Representative fluorescence microscope images of hermaphrodites that contain a DAF-16::GFP transgene. Animals were cultured with control RNAi (upper panels) or *acn-1* RNAi (lower panels). Animals were cultured at 20°C continuously (left panels) or heat shocked by exposure to 35°C for 30 minutes (right panels). In standard culture conditions, DAF-16::GFP was not nuclear localized (left panels). By contrast, heat shock caused nuclear localization of DAF-16::GFP (right panels, red arrows indicate fluorescent nuclei). Animals treated with *acn-1* RNAi were similar to animals treated with control RNAi.
(TIF)

S1 Table *acn-1* RNAi and Captopril treatment did not cause substantial nuclear localization of DAF-16::GFP ^1^ RNAi: Animals were cultured at 20°C with bacteria containing the control RNAi plasmid (L4440) or the *acn-1* and / or *daf-2* RNAi plasmid starting at the embryonic stage. ^2^Drug: Animals were cultured on standard NGM (None) or with NGM containing 2.5mM captopril starting at the L4 stage. ^3^Animals were transferred for 30 min to 35°C, a heat stress. ^4^GFP was diffusely localized in the cytosol. ^5^GFP localization was defined as “nuclear” if most or all nuclei displayed intensely fluorescing puncta throughout the entire body from head to tail, or defined as “intermediate” if puncta were observed in at least one or more nuclei but not most or all nuclei. ^6^N: Number of hermaphrodites analyzed.
(DOCX)
