# Supplementary figures and images for: Angiotensin Converting Enzyme (ACE) Inhibitor Extends Caenorhabditis elegans Life Span
Source: PLoS Genet. 2016 Feb 26;12(2):e1005866. doi: 10.1371/journal.pgen.1005866 (PMC4769152; doi:10.1371/journal.pgen.1005866)

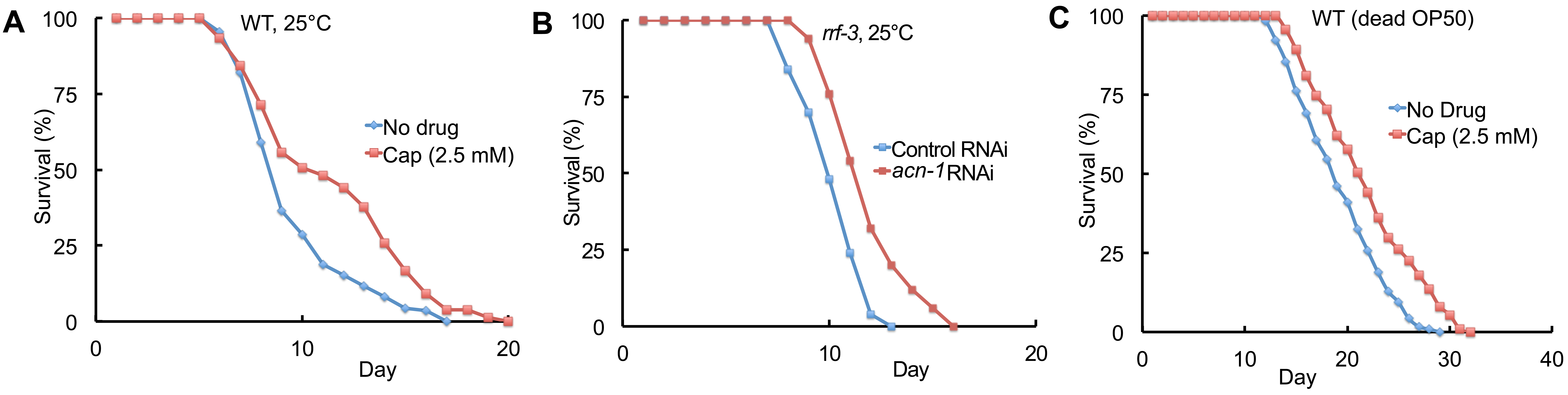

Supplement: S1 Fig — (A) Survival curves of wild-type (WT) hermaphrodites cultured at 25°C with no drug or 2.54 mM captopril (Cap) in the NGM medium. Hermaphrodites were exposed to captopril starting at the L4 stage (day 0) and monitored regularly until death. See Table 1 for summary statistics, number of animals and number of independent experiments. (B) Survival curves of rrf-3 mutant hermaphrodites cultured at 25°C with bacteria containing the control RNAi plasmid (L4440, blue) or the acn-1 RNAi plasmid (red). Hermaphrodites were exposed to RNAi bacteria starting at the embryonic stage. See Table 2 for summary statistics. These data represent a single experiment (N = 50). (C) Survival curves of wild-type (WT) hermaphrodites cultured at 20°C with E. coli OP50 that was killed by exposure to ultraviolet light with no drug or 2.54 mM captopril (Cap) in the NGM medium. Hermaphrodites were exposed to captopril starting at the L4 stage (day 0) and monitored regularly until death. See Table 1 for summary statistics, number of animals and number of independent experiments. (TIF) [file pgen.1005866.s001.tif]

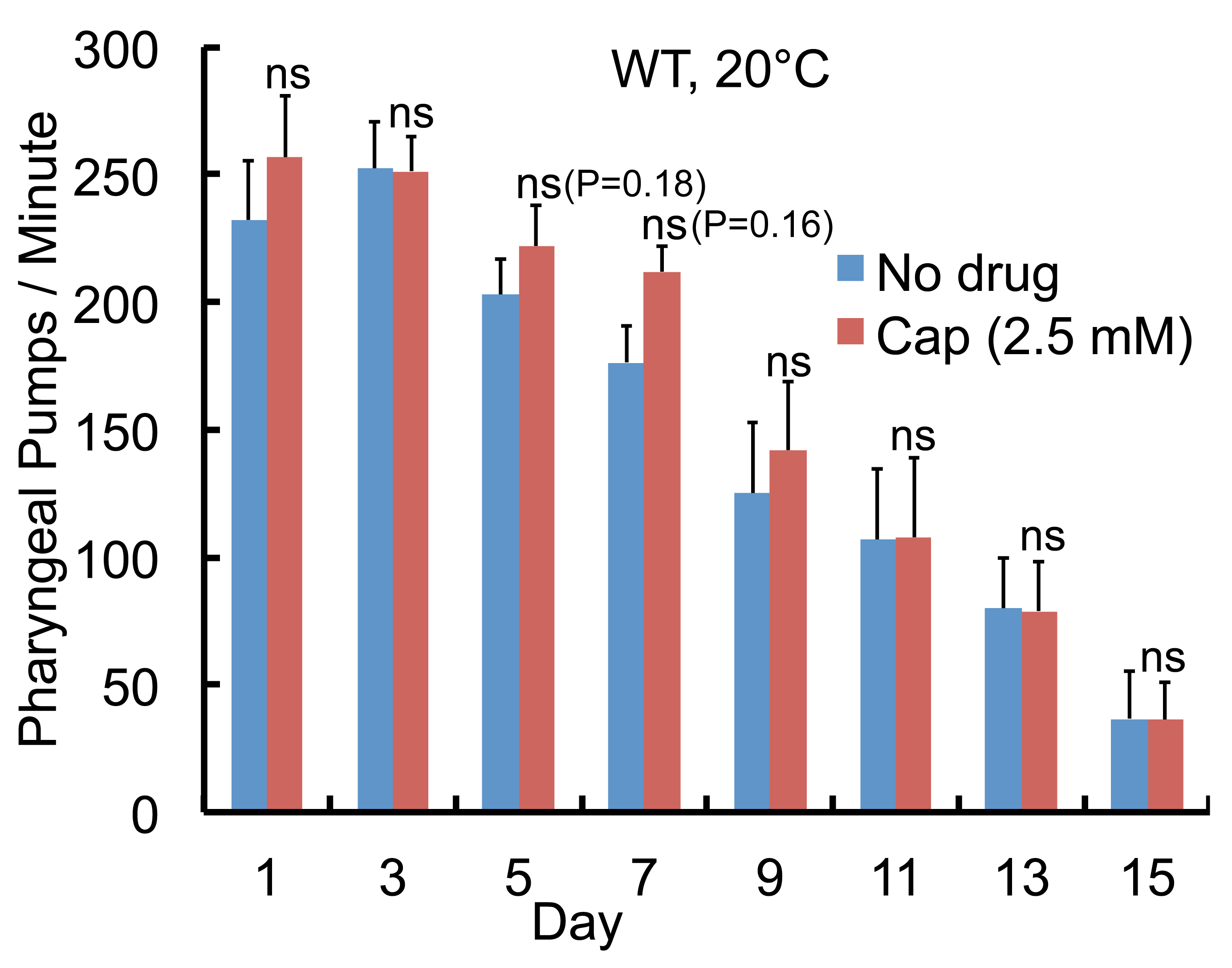

Supplement: S2 Fig — Bars show the pharyngeal pumping rate in beats per minute and the standard deviation. The rate was measured by counting beats for 10 seconds using a dissecting microscope. Wild-type animals were treated with no drug (blue) or 2.54 mM captopril (red) starting at the L4 stage (day 0) and cultured at 20°C (N = 25). n.s., not significant, P > 0.05. Captopril treated animals displayed a small increase in pumping rate at days 5–9, but this trend was not statistically significant with this sample size. (TIF) [file pgen.1005866.s002.tif]

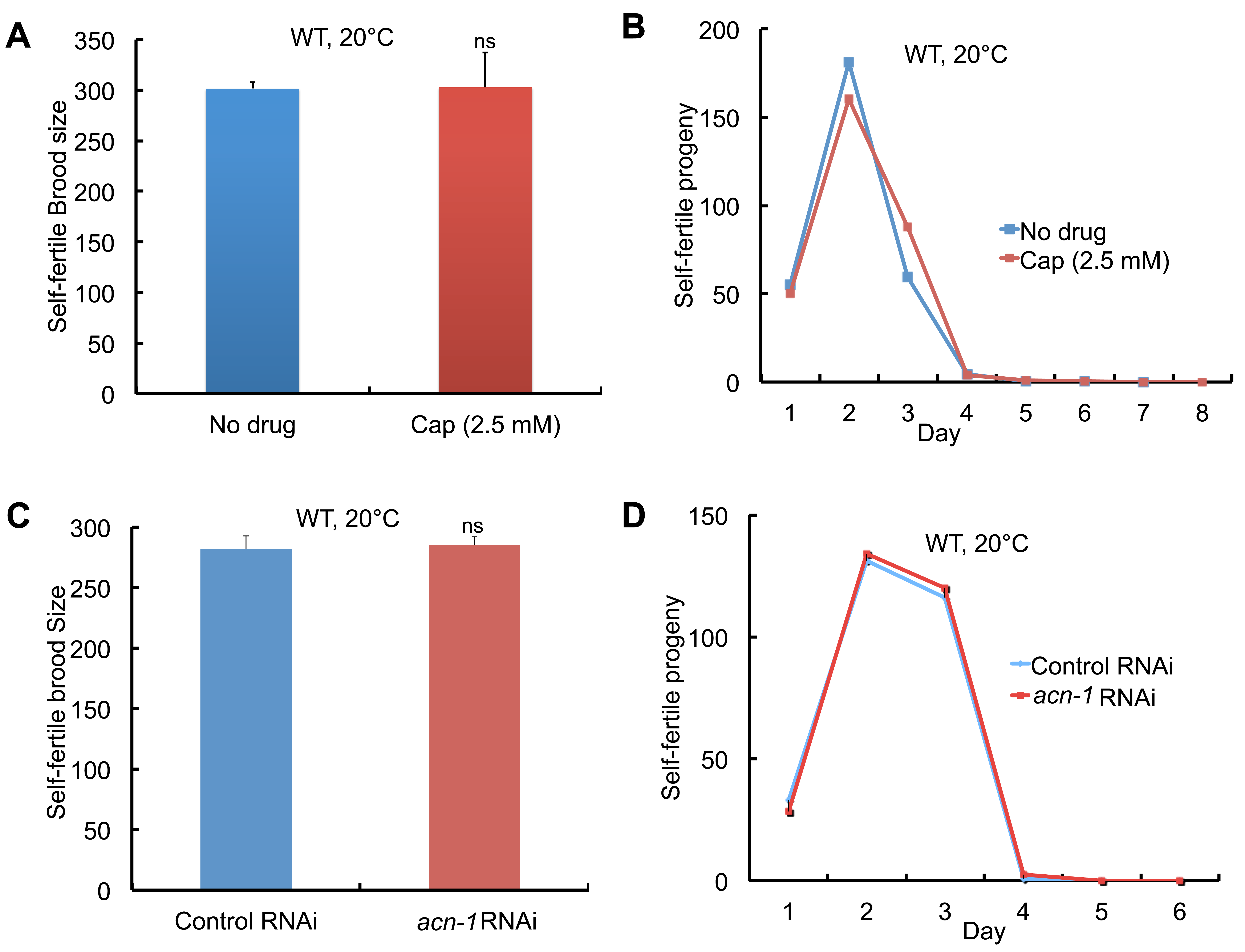

Supplement: S3 Fig — Wild type self-fertile hermaphrodites were cultured at 20°C and treated with (A, B) no drug (blue) or 2.54 mM captopril (red) starting at the L4 stage (day 0). (C, D) Animals were cultured with RNAi bacteria containing the control RNAi plasmid (L4440, blue) or the acn-1 RNAi plasmid (red). Hermaphrodites were exposed to RNAi bacteria starting at the embryonic stage. (A, C) Bars show total number of live progeny and standard deviation. (B, D) Data points show total number of live progeny produced each day. Number of animals analyzed: no drug (N = 3), captopril (N = 3), Control RNAi (N = 5) and acn-1 RNAi (N = 5). n.s., not significant, P > 0.05. (TIF) [file pgen.1005866.s003.tif]

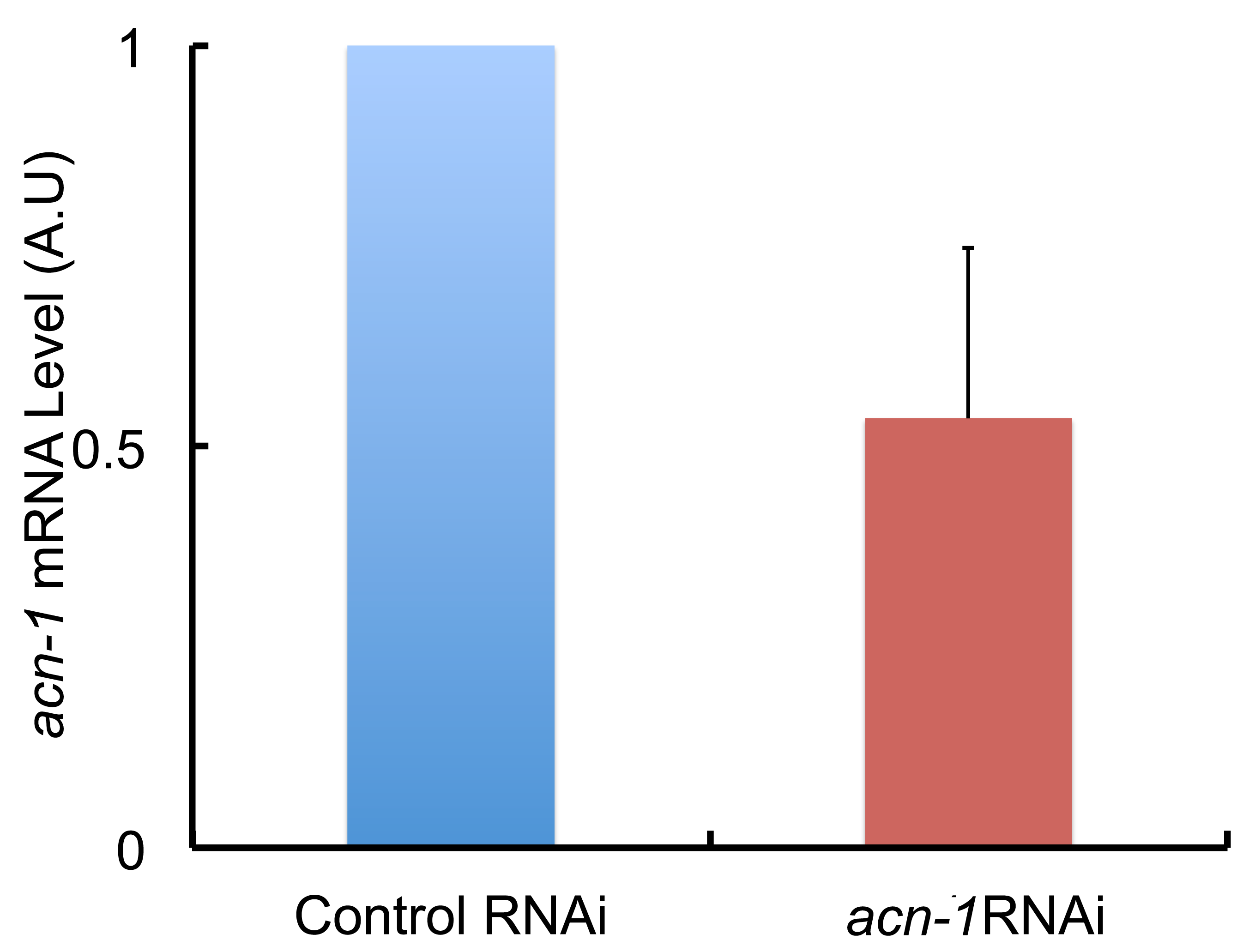

Supplement: S4 Fig — mRNA was isolated from populations of two day old adult rrf-3(pk1426) animals cultured with control RNAi (blue) or acn-1 RNAi (red). acn-1 transcript levels were analyzed by RT-PCR; mRNA levels are expressed in arbitrary units (A.U.) and were normalized to rps-23, a ribosomal protein. The values were normalized by setting the value for control RNAi equal to 1.0. Bars represent the average +/- S.E.M. (n = 3 biological replicates). (TIF) [file pgen.1005866.s004.tif]

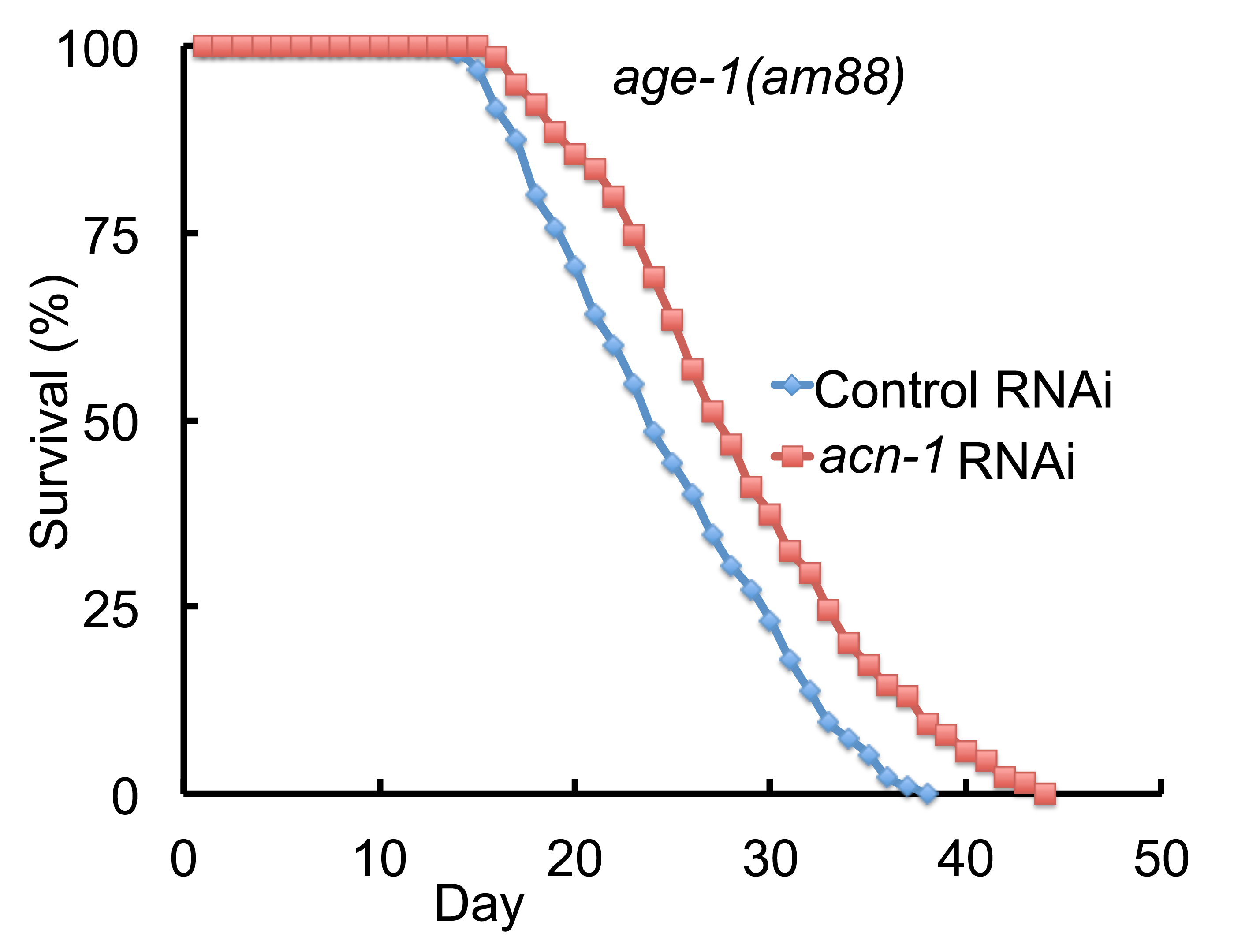

Supplement: S5 Fig — Survival curves of age-1(am88) mutant hermaphrodites cultured at 20°C with bacteria containing the control RNAi plasmid (L4440, blue) or the acn-1 RNAi plasmid (red). Hermaphrodites were exposed to RNAi bacteria starting at the embryonic stage and monitored regularly until death. See Table 2 for summary statistics, number of animals and number of independent experiments. (TIF) [file pgen.1005866.s005.tif]

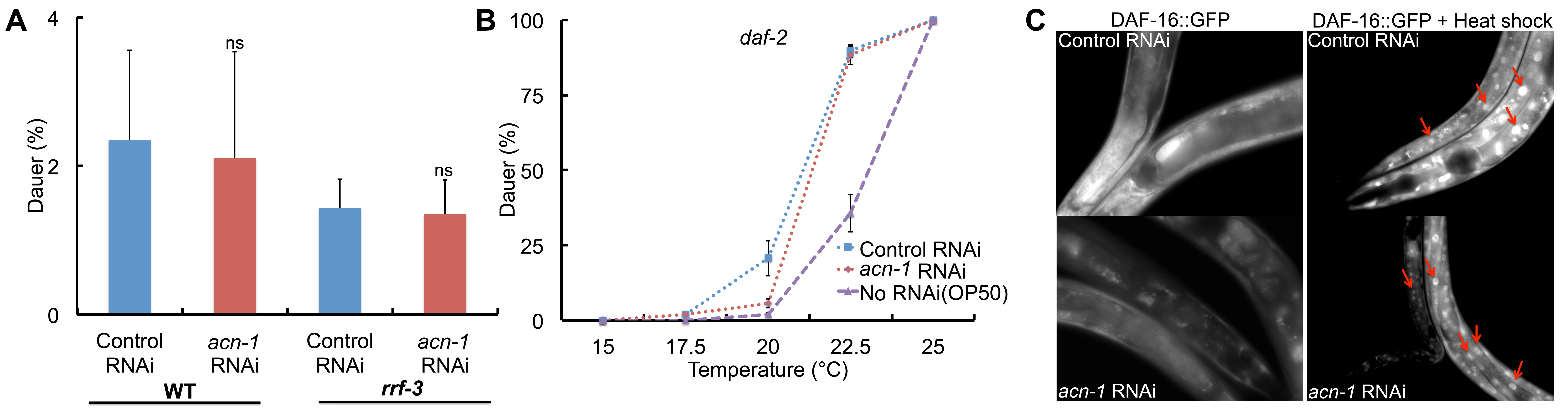

Supplement: S6 Fig — Bars (A) and data points (B) indicate the percent of embryos that formed dauer larvae and standard deviation. Adult hermaphrodites were cultured at 20°C with bacteria containing the control RNAi plasmid (L4440, blue) or the acn-1 RNAi plasmid (red). Embryos were cultured for three days at 27°C (A) or the indicated temperature (B) and scored for dauer larvae formation based on morphological criteria using a dissecting microscope. (A) Genotypes were wild type and rrf-3(pk1426). (B) daf-2 (e1370) caused a temperature sensitive Daf-c phenotype when cultured with no RNAi (E. coli OP50 bacteria) (purple triangles). Control RNAi and acn-1 RNAi both increased the penetrance of the Daf-c phenotype to a similar extent, indicating that the effect is caused by the bacterial strain used for RNAi rather than the inhibition of the acn-1 gene. Comparisons are to the paired control RNAi: n.s., not significant, P > 0.05. (C) Representative fluorescence microscope images of hermaphrodites that contain a DAF-16::GFP transgene. Animals were cultured with control RNAi (upper panels) or acn-1 RNAi (lower panels). Animals were cultured at 20°C continuously (left panels) or heat shocked by exposure to 35°C for 30 minutes (right panels). In standard culture conditions, DAF-16::GFP was not nuclear localized (left panels). By contrast, heat shock caused nuclear localization of DAF-16::GFP (right panels, red arrows indicate fluorescent nuclei). Animals treated with acn-1 RNAi were similar to animals treated with control RNAi. (TIF) [file pgen.1005866.s006.tif]
