# Supplementary material for: Angiotensin Converting Enzyme (ACE) Inhibitor Extends Caenorhabditis elegans Life Span
Source: PLoS Genet. 2016 Feb 26;12(2):e1005866. doi: 10.1371/journal.pgen.1005866 (PMC4769152; doi:10.1371/journal.pgen.1005866)
Supplement: S1 Table — 2Drug: Animals were cultured on standard NGM (None) or with NGM containing 2.5mM captopril starting at the L4 stage. 3Animals were transferred for 30 min to 35°C, a heat stress. 4GFP was diffusely localized in the cytosol. 5GFP localization was defined as “nuclear” if most or all nuclei displayed intensely fluorescing puncta throughout the entire body from head to tail, or defined as “intermediate” if puncta were observed in at least one or more nuclei but not most or all nuclei. 6N: Number of hermaphrodites analyzed. (DOCX) [file pgen.1005866.s007.docx]

| **RNAi^1^** | **Drug^2^** | **Heat^3^ shock**  **(35°C)** | **Cytosolic^4^**  **(%)** | **Intermediate^5^ /nuclear (%)** | **N^6^** |
| --- | --- | --- | --- | --- | --- |
| Control | None | - | 92.6 | 7.4 | 27 |
| Control | None | + | 16.7 | 83.3 | 24 |
| *acn-1* | None | - | 87 | 13 | 30 |
| *acn-1* | None | + | 14 | 86 | 43 |
| *daf-2* | None | - | 15.6 | 84.4 | 45 |
| Control+ *daf-2* | None | - | 17.9 | 82.1 | 39 |
| *acn-1*+*daf-2* | None | - | 12 | 88 | 25 |
| None | Captopril (2.54 mM) | - | 95.2 | 4.7 | 21 |
| Control | Captopril (2.54 mM) | - | 94.1 | 5.8 | 17 |
| *daf-2* | Captopril (2.54 mM) | - | 10 | 90 | 30 |

**S1 Table *acn-1* RNAi and Captopril treatment did not cause substantial nuclear localization of DAF-16::GFP**
